# The adrenergic-induced ERK3 pathway drives lipolysis and suppresses energy dissipation

**DOI:** 10.1101/gad.333617.119

**Published:** 2020-04-01

**Authors:** Rabih El-Merahbi, Jonathan Trujillo Viera, Angel Loza Valdes, Katarzyna Kolczynska, Saskia Reuter, Mona C. Löffler, Manuela Erk, Carsten P. Ade, Till Karwen, Alexander E. Mayer, Martin Eilers, Grzegorz Sumara

**Affiliations:** 1 Rudolf Virchow Center for Experimental Biomedicine, University of Würzburg, 97080 Würzburg, Germany;; 2 Nencki Institute of Experimental Biology, PAS, 02-093 Warsaw, Poland;; 3 Theodor Boveri Institute, Biocenter, University of Würzburg, Am Hubland, 97074 Würzburg, Germany

**Keywords:** lipolysis, ATGL/ERK3, obesity, MK5, FOXO1, PKA, adrenalin, UCP1

## Abstract

Obesity-induced diabetes affects >400 million people worldwide. Uncontrolled lipolysis (free fatty acid release from adipocytes) can contribute to diabetes and obesity. To identify future therapeutic avenues targeting this pathway, we performed a high-throughput screen and identified the extracellular-regulated kinase 3 (ERK3) as a hit. We demonstrated that β-adrenergic stimulation stabilizes ERK3, leading to the formation of a complex with the cofactor MAP kinase-activated protein kinase 5 (MK5), thereby driving lipolysis. Mechanistically, we identified a downstream target of the ERK3/MK5 pathway, the transcription factor FOXO1, which promotes the expression of the major lipolytic enzyme ATGL. Finally, we provide evidence that targeted deletion of ERK3 in mouse adipocytes inhibits lipolysis, but elevates energy dissipation, promoting lean phenotype and ameliorating diabetes. Thus, ERK3/MK5 represents a previously unrecognized signaling axis in adipose tissue and an attractive target for future therapies aiming to combat obesity-induced diabetes.

## Abstract (precis)

In this study, El-Merahbi et al. investigated new regulators of lipolysis, and using a high-throughput screen identified the extracellular-regulated kinase 3 (ERK3) in lipolysis regulation. They identified a downstream target of the ERK3/MK5 pathway, the transcription factor FOXO1, which promotes expression of the major lipolytic enzyme ATGL, and provide evidence that targeted deletion of ERK3 in mouse adipocytes inhibits lipolysis.

Adaptation to changes in nutrient availability is pivotal for the survival of living organisms. Adipose tissue is a central storage organ in the body. Upon nutrient ingestion, insulin promotes triglycerides (TG) synthesis and storage in the adipocytes. During food deprivation or increased energy demand, lipolysis of triglycerides (TG) stored in lipid droplets of adipocytes provides peripheral tissues with essential nutrients: free fatty acids (FFAs) and glycerol. Consequently, this leads to the reduction of adipose tissue mass. However, uncontrolled induction of lipolysis independently of the organism's nutritional demands is also associated with the development of multiple metabolic diseases including type 2 diabetes and cancer-associated cachexia (Haemmerle et al. 2006; Das et al. 2011; Arner and Langin 2014; Schreiber et al. 2015). Paradoxically, inhibition of specific components of lipolytic machinery can ameliorate diet and genetically induced obesity (Sekiya et al. 2004; Schreiber et al. 2015; Schweiger et al. 2017), indicating that induction of lipolysis can result in different physiological outcomes depending on the nutritional status of the organism. However, the signaling pathways that link the nutritional and hormonal status with temporarily controlled lipolytic response remain obscure.

So far, several hormones are known to regulate the rate of lipolysis in response to different physiological challenges. Catecholamines, acting through the β3-adrenergic receptor, represent major endocrine factors inducing TG degradation (Zechner et al. 2012). Recently, we proposed that gut-derived serotonin promotes lipolysis by acting through the HTR2B receptor (Sumara et al. 2012; El-Merahbi et al. 2015). These and other hormones, as well as several inflammatory mediators, activate assembly of the lipolytic machinery in the lipid droplets (Zechner et al. 2012). In contrast, insulin-induced signaling attenuates expression and activity of lipolytic enzymes and inhibits lipolysis rate (Zechner et al. 2017).

In the lipid droplets, three enzymes were described to mediate lipolysis: adipose triglyceride lipase (ATGL, also known as patatin-like phospholipase domain-containing protein 2), hormone-sensitive lipase (HSL), and monoglyceride lipase (MGL) (Zechner et al. 2012). As revealed by mouse genetic experiments, deletion of ATGL results in almost complete inhibition of FFAs and glycerol release, while silencing of HSL or MGL in adipose tissue have a moderate effect on lipolysis rate in mice (Osuga et al. 2000; Haemmerle et al. 2002, 2006; Taschler et al. 2011). Additionally, the rate of lipolysis is regulated by a number of lipid droplet-associated proteins including perilipins, CGI-58, and G0S2, which are required for efficient stimulation of lipolysis (Zechner et al. 2017).

However, signaling events inducing lipolysis in adipocytes in response to the catecholamines and serotonin stimulation remain poorly characterized. Activation of protein kinase A (PKA) is one known signaling event required for induction of lipolytic enzymes upon activation of the β3-adrenergic receptor. PKA directly phosphorylates HSL to induce its activity (Zechner et al. 2017). It also phosphorylates perilipin and CGI-58, which allows CGI-58 to get into a complex with ATGL and to activate this lipase (Zechner et al. 2017). However, how PKA can mediate the signaling maintaining the transcription of the lipolytic machinery in response to hormonal stimulation is not understood.

Therefore, we designed an unbiased screen to identify the factors that could control lipolysis. We reasoned that such a response is likely to occur rapidly through posttranslational mechanisms and chose to screen through genes encoding for all known protein kinases. Unexpectedly, we identified a high number of kinases potentially implicated in the regulation of lipolysis. Among them, silencing of *Erk3* resulted in the highest suppression of lipolysis rate. ERK3 (also known as MAPK6) is an atypical member of the MAPK family. ERK3 is a constitutively active kinase; therefore, its abundance determines the rate of substrates phosphorylation (Coulombe et al. 2003, 2004). In quiescent cells, ERK3 is subjected to rapid proteasome-mediated degradation (Coulombe et al. 2003, 2004). Interestingly, we demonstrated that β-adrenergic-induced PKA signaling stabilizes ERK3 by promoting the formation of the complex between ERK3 and MAP kinase-activated protein kinase 5 (MK5), which protects both kinases from degradation. Moreover, we demonstrated that ERK3/MK5 pathway activates the translocation of Forkhead box protein O1 (FOXO) to the nucleus, which promotes ATGL expression. Consistently, the deletion of *Erk3* in adipose tissue or inhibition of MK5 in mice results in a decrease of *Atgl* expression and lipolysis. Surprisingly, mice deficient for *Erk3* specifically in adipocytes are resistant to diet-induced obesity and diabetes but display elevated energy expenditure, suggesting that the balance between the nutritional demands and lipolysis rate is perturbed in the absence of ERK3. We propose that the ERK3/MK5 pathway represents a missing link downstream from PKA required for the fine-tuning of the lipolytic transcriptional signaling and an attractive target for future antiobesity and antidiabetic therapies.

## Results

### siRNA-based screen in adipocytes reveals ERK3 as a central regulator of lipolysis

**Figure 1.**
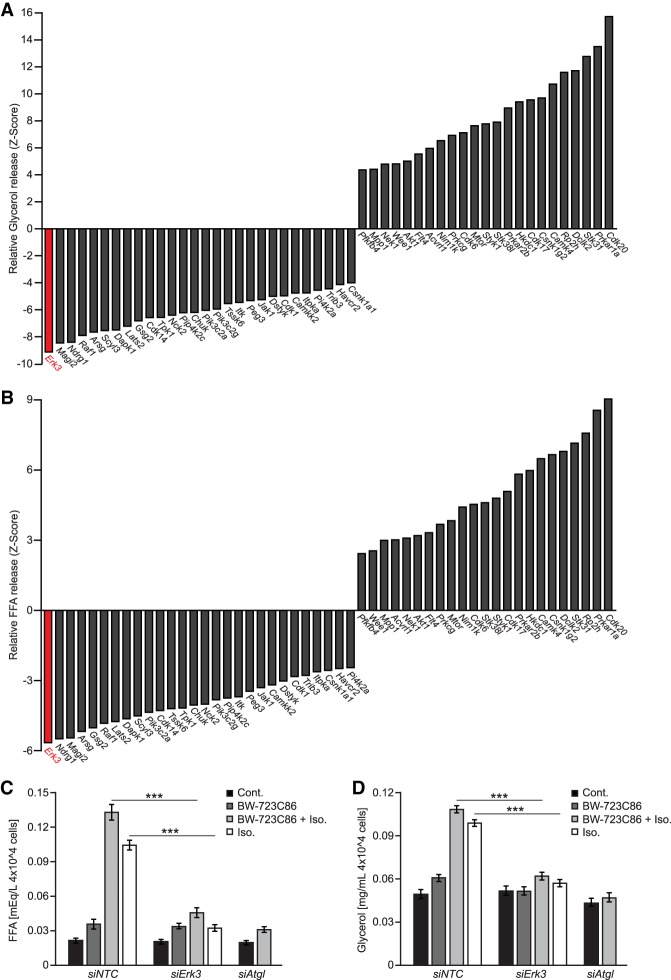
ERK3 is required for induction of lipolysis. (*A*,*B*) Relative rate of glycerol release (*A*) and FFA release (*B*) (Z-score; *n* = 4) from 3T3L1 cells transfected with the indicated siRNA pools. (siNTC) Nontargeting control. (*C*,*D*) FFA (*C*) and glycerol (*D*) release from stromal–vascular cell (SVC)-derived adipocytes transfected with specific siRNAs and stimulated with the β-agonist isoproterenol (Iso.) and/or the HTR2b agonist BW-723C86 as indicated (*n* = 3). Data are presented as average ± SEM, (***) *P* ≤ 0.001.

We designed a screening strategy to assess the impact of kinase-mediated signaling on the rate of lipolysis evoked by the β-adrenergic agonist, isoproterenol (Iso.), and the HTR2B agonist, BW-723C86, in differentiated adipocyte-like cells 3T3L1 (Supplemental Fig. S1a). Cotreatment of adipocytes with Iso. and BW-723C86 resulted in maximal stimulation of glycerol and FFAs release (Supplemental Fig. S1c,d). We verified our screening strategy using siRNA-mediated silencing of ATGL. Indeed, depletion of ATGL resulted in a strong reduction of FFAs and glycerol release from adipocytes (Supplemental Fig. S1b–d). The primary screen revealed that silencing of 48 kinases resulted in decreased lipolysis (FFAs output), whereas depletion of 69 kinases enhanced it in 3T3L1-derived adipocytes (Supplemental Table 1). In a secondary screen (using a different set of siRNA pools) we confirmed that silencing of 28 kinases reduced glycerol and FFA release, while silencing of 23 enhanced it (Fig. 1A,B). Of note, *Prkar1a*, *Prkar2b*, *Ndrg1*, *Raf1*, *Lats2*, *Peg3*, and *Trib3*, which appeared in our screen, were previously implicated in the regulation of lipolysis and different aspects of adipocytes function (Curley et al. 2005; Qi et al. 2006; Kalderon et al. 2012; An et al. 2013; Cai et al. 2017; Torres-Quesada et al. 2017). These confirm the accuracy of our screening strategy.

Interestingly, silencing of Extracellular regulated kinase 3 (*Erk3*) inhibited lipolysis to the largest extent in both screens (Fig. 1A,B; Supplemental Table 1). Consistently, silencing of *Erk3* in adipocytes derived from primary stromal vascular cells or 3T3L1 cells (by specific siRNAs and shRNA) resulted in almost complete suppression of glycerol and FFAs release evoked by β-agonists and HTR2B agonists (Fig. 1C,D; Supplemental Fig. S2C–G) comparable with the silencing of *Atgl* (Fig. 1C,D; Supplemental Fig. S2C,D).

### β-Adrenergic activation of PKA leads to stabilization of ERK3

**Figure 2.**
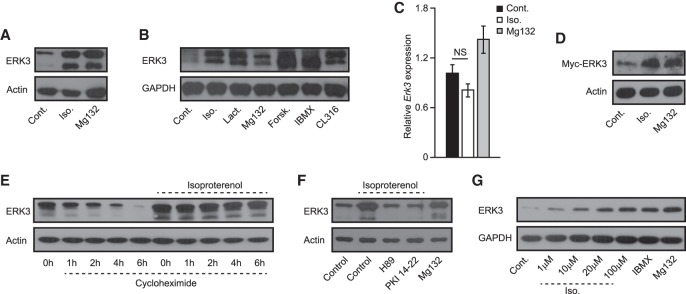
β-Adrenergic signaling promotes ERK3 protein levels in a PKA-dependent manner. (*A*,*B*,*F*) Western blot for ERK3 on differentiated 3T3L1 cells stimulated as indicated for 2 h. (Iso.) Isoproterenol; (proteasome inhibitor) Mg132 and lactacystin; (cAMP elevators Forsk.) Forskolin and IBMX; (PKA inhibitors) H89 and PKI 14-22. (*C*) Relative expression of Erk3 in 3T3L1 cells stimulated as shown for 2 h. (*D*) Western blot analysis of indicated proteins on 3T3L1-derived adipocytes expressing Myc-tagged ERK3 and stimulated as shown for 2 h. (*E*) ERK3 levels on 3T3L1 cell treated with cycloheximide for indicated time points and stimulated with Iso. for 2 h prior to cycloheximide treatment (*G*) Western blot analysis of indicated proteins on Hek293T cells stimulated as indicated for 2 h. *n* = 3 for each experiment. For graphs, data are presented as average ± SEM.

In quiescent cells, ERK3 is subjected to rapid proteasome-mediated degradation (Coulombe et al. 2003, 2004). Consistent with this, incubation of adipocytes with proteasome inhibitors (Mg132 or lactacystin) stabilized ERK3 (Fig. 2A,B). In addition, incubation of adipocytes with β-adrenergic agonists (Iso. and CL316) also increased ERK3 levels (Fig. 2A,B), while mRNA levels of *Erk3* were unaffected (Fig. 2C). The abundance of ectopically expressed Myc-tagged ERK3 was also stabilized by the β-agonist and the proteasome inhibitor (Fig. 2D). Finally, blocking translation in adipocytes with cycloheximide decreased ERK3 levels over time, which was inhibited by Iso. (Fig. 2E). This demonstrates that β-agonists stabilize ERK3 at the protein level, likely via inhibiting its proteasomal degradation.

We next sought to identify the mechanism by which β-adrenergic agonists stimulate ERK3 levels and promote lipolysis in adipocytes. β-Agonists promote cAMP levels in cells, thereby activating protein kinase A (PKA) (Arner and Langin 2014). Indeed, the elevation of cAMP levels independently of β-adrenergic receptors (by forskolin or IBMX) was sufficient to stabilize ERK3 (Fig. 2B). Moreover, inhibition of PKA with either H89 or PKI 14-22 prevented β-agonist-induced stabilization of ERK3 in adipocytes (Fig. 2F). Additionally, stimulation with β-agonists, compounds promoting cAMP levels, or treatment with proteasomal inhibitors also stabilized ERK3 in Hek293 and undifferentiated 3T3L1 cells (Fig. 2G; Supplemental Fig. S3a). Thus, β-adrenergic stimulation promotes PKA-dependent ERK3 stabilization.

### PKA-mediated phosphorylation of MK5 promotes ERK3 stabilization and lipolysis

**Figure 3.**
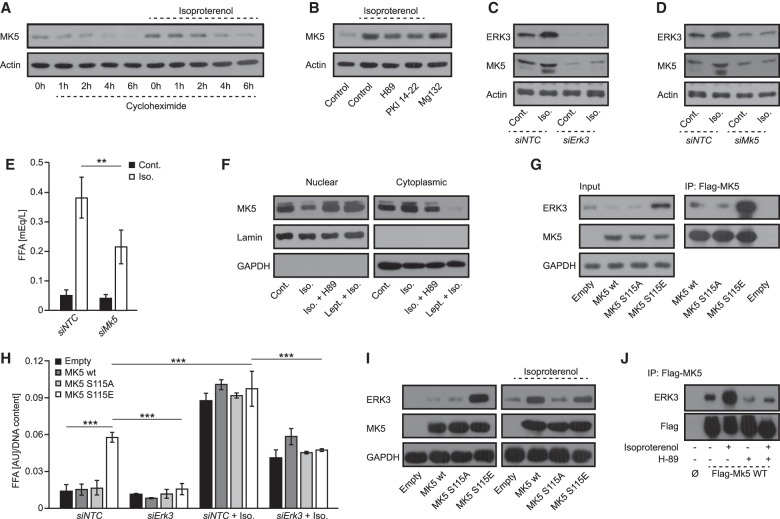
Stabilization of ERK3 by β-adrenergic stimulation requires PKA-dependent phosphorylation of MK5. (*A*) Western blot analysis for MK5 and actin after blockage of translation by cycloheximide for indicated time points, cells were pretreated with isoproterenol or vehicle for 2 h before the addition of cycloheximide. (*B*) Western blot analysis for MK5 on differentiated 3T3L1 cells stimulated as indicated (cycloheximide, isoproterenol, Mg132, and PKA inhibitors H89 and PKI 14-22) for 2 h. (*C*,*D*) Western blot analysis on ERK3 and MK5 protein levels in 3T3L1 adipocytes transfected with *Erk3*- or *Mk5*-specific siRNA respectively and compared with nontargeting control transfected cells. Cells were treated with vehicle or isoproterenol for 2 h before collection. (*E*) FFAs release from *Mk5*-depleted (siMk5) and nontargeting control (siNTC) 3T3L1 cells in response to stimulation with isoproterenol for 2 h. (*F*) MK5 levels in cytoplasmic and nuclear fractions of 3T3L1 cells treated as indicated for 2 h. (Lept) Leptomycin B. (*G*, *left* panel, input) ERK3 and MK5 levels in adipocytes transduced with MK5 mutants as shown. (*Right* panel) Immunoprecipitation (IP) using Flag antibody of indicated Flag-tagged mutants of MK5, followed by Western blot using specified antibodies against coprecipitated proteins. (*H*) FFA release from adipocytes expressing indicated MK5 mutants and transfected with specific siRNAs and treated as shown (for 2 h). (*I*) Western blot for indicated proteins on extracts isolated from cells expressing indicate mutants of MK5 stimulated as shown for 2 h. (*J*) IP using Flag antibody on extracts prepared from cells stimulated as indicated for 2 h, followed by Western blot for ERK3 and Flag. *n* = 3 for each experiment. For graphs, data are presented as average ± SEM, (**) *P* ≤ 0.01; (***) *P* ≤ 0.001.

Formation of a complex between ERK3 and MK5 protects both kinases from degradation and leads to the activation of MK5 (Schumacher et al. 2004; Seternes et al. 2004). It has been also shown that PKA phosphorylates MK5 at Ser115 and might regulate the subcellular localization of MK5 (Kostenko et al. 2011). Interestingly, our primary screen revealed that silencing of *Mk5* reduced the rate of lipolysis (Supplemental Table 1). Therefore, we hypothesized that a PKA/ERK3/MK5 signaling axis may regulate lipolysis induced by β-adrenergic stimulation. Consistent with this hypothesis, we found that β-adrenergic stimulation also stabilized *Mk5* (Fig. 3A), which was partially blocked by PKA inhibition (Fig. 3B), as shown previously for ERK3 (Fig. 2F). Silencing of *Erk3* in adipocytes decreased the abundance of MK5 (Fig. 3C), while mRNA levels were unaffected (Supplemental Fig. S3b). Similarly, silencing of MK5 resulted in decreased abundance of ERK3 but did not affect its expression (Fig. 3D; Supplemental Fig. S3c). Consistently, silencing of *Mk5* significantly reduced FFAs and glycerol output from adipocytes upon β-agonist stimulation (Fig. 3E; Supplemental Fig. S3d). Thus, β-adrenergic-stimulated MK5 also promotes lipolysis in adipocytes.

PKA-dependent phosphorylation of MK5 promotes its translocation from the nucleus to the cytoplasm (Kostenko et al. 2011). This raises the possibility that, upon adipocyte stimulation, PKA-dependent phosphorylation of MK5 might allow for the formation of a complex between MK5 and ERK3 as well as stabilization of both kinases, and thereby induction of lipolysis. Consistent with this, stimulation of adipocytes with β-agonist resulted in redistribution of MK5 from the nucleus to the cytoplasm, which was abolished by PKA inhibition (Fig. 3F). Importantly, leptomycin B, which blocks nuclear export, prevented β-agonist-induced ERK3 stabilization (Fig. 3F; Supplemental Fig. S3e), indicating that translocation of MK5 to the cytoplasm is required for ERK3 stabilization. To test this directly, we generated phospho-deficient and phospho-mimetic mutants of MK5 on Ser115 (MK5-S115A and MK5-S115E, respectively). MK5-S115A localized primarily to the nucleus, while MK5-S115E displayed cytoplasmic localization (Supplemental Fig. S3f). Importantly, MK5-S115E mutation promoted the formation of a complex with endogenous ERK3 (Fig. 3G). Consistently, expression of MK5-S115E, but not wild-type MK5 or MK5-S115A, was sufficient to stabilize ERK3 (Fig. 3G,I) and evoke lipolysis even in the absence of β-agonist (Fig. 3H). Of note, the abundance and expression of different MK5 mutants were equal (Fig. 3G; Supplemental Fig. S3g). Ectopic expression of MK5 mutants also did not affect ERK3 at the transcriptional level (Supplemental Fig. S3h). Importantly, in cells stimulated with β-adrenergic agonist, overexpression of the wild-type form of MK5 was sufficient to stabilize ERK3 to a higher extent than in the control cells (Fig. 3I). Finally, β-adrenergic stimulation of adipocytes promoted the formation of the complex between MK5 and ERK3, while inhibition of PKA activity blocked this interaction (Fig. 3J). Taken together, these results indicate that PKA-dependent phosphorylation of MK5 on Ser115 is required to stimulate MK5 nuclear export and a complex formation with ERK3, which results in stabilization of both kinases and induction of lipolysis.

### The ERK3/MK5 pathway promotes nuclear translocation of FOXO1 to drive *Atgl* expression

**Figure 4.**
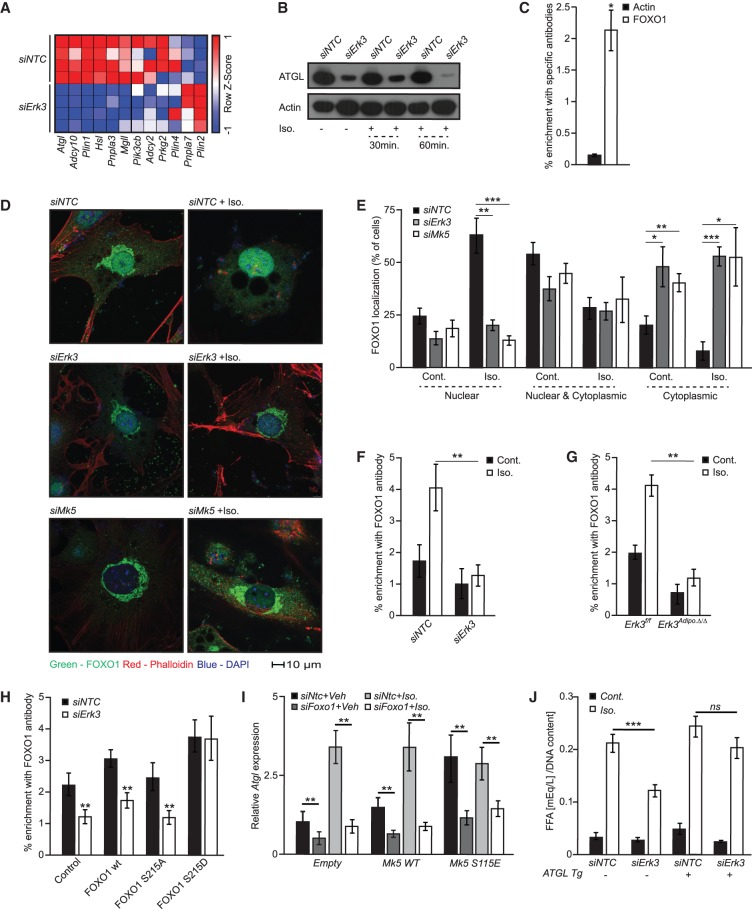
ERK3/MK5 promotes FOXO1-mediated transcription of Atgl. (*A*) RNA sequencing-based heat map of the expression patterns of indicated genes. (*B*) Western blot for ATGL on 3T3L1 adipocytes transfected with control or Erk3 siRNA and treated with Iso. for 30 or 60 min. (*C*) Chromatin immunoprecipitation of *Atgl* promoter using FOXO1 and actin antibody from 3T3L1 (*D*,*E*) Representative images of FOXO1 staining on 3T3L1 cells transfected with indicated siRNAs, stimulated as shown for 2 h and corresponding quantifications of specific localizations. (*F*–*H*) Chromatin immunoprecipitation of *Atgl* promoter using FOXO1 antibody from 3T3L1 cells transfected with Erk3 or NTC siRNA (*F*) and SVC-derived brown adipocytes (*G*) stimulated for 2 h as shown, or expressing specific FOXO1 mutants (*H*). (*I*) Relative expression of *Atgl* in 3T3L1 cells expressing the indicated mutants of MK5, transfected with NTC or *Foxo1*-specific siRNA and stimulated with Iso. for 2 h. (*J*) FFAs release from 3T3L1 cells ectopically expressing *Atgl* (Atgl tg), transfected with NTC or *Erk3*-specific siRNA and stimulated with Iso. for 2 h. *N* = 3–4 for each experiment. For graphs, data are presented as average ± SEM. (*) *P* ≤ 0.05; (**) *P* ≤ 0.01; (***) *P*≤0.001.

To identify the mechanism by which ERK3/MK5 promotes lipolysis, we subjected control and ERK3-deficient adipocytes to RNA sequencing. Global transcriptomic analysis revealed that ERK3 is required for expression of genes inducing lipolysis (Fig. 4A; Supplemental Fig. S4a). Consistently, qPCR confirmed the reduced expression of the major lipolytic enzyme *Atgl* (Zechner et al. 2012) in adipocytes depleted of *Erk3* or *Mk5* (Supplemental Fig. S4b). Similarly, ATGL protein was reduced in adipocytes depleted of *Erk3* (Fig. 4B).

Forkhead box protein O family members are transcription factors that function broadly in regulating energy homeostasis, including insulin and glucose metabolism (Eijkelenboom and Burgering 2013). FOXO proteins can be phosphorylated by MK5, and FOXO1 was proposed to promote ATGL expression (Chakrabarti and Kandror 2009b; Kress et al. 2011; Chow et al. 2013). Consistently, we showed that FOXO1 binds to the *Atgl* promoter in adipocytes (Fig. 4C), and depletion of FOXO1 reduced *Atgl* expression as well as lipolysis (Supplemental Fig. S4b,c). MK5 phosphorylates members of the FOXO family on Ser215, which is conserved among all the FOXO members (including FOXO1) (Kress et al. 2011; Chow et al. 2013). We have generated phospho-deficient and phospho-mimetic mutants of FOXO1 (FOXO1 S215A and FOXO1 S215D, respectively). Importantly, FOXO1 S215A displayed primarily cytoplasmic localization while FOXO1 S215D localized mainly in the nucleus (Supplemental Fig. S4d). Silencing of either *Erk3* or *Mk5* in adipocytes reduced the nuclear localization of FOXO1 (Fig. 4D,E). Additionally, in ERK3-deficient white and brown adipocytes, we observed reduced binding of FOXO1 to the *Atgl* promoter (Fig. 4F,G; Supplemental Fig. S4e). Moreover, expression of the phospho-mimetic FOXO1 mutant (S215D), but not wild-type or phospho-deficient (S215A) FOXO1, restored binding to the *Atgl* promoter in adipocytes depleted of ERK3 (Fig. 4H). To further assess whether FOXO1 mediates β-adrenergic and ERK3/MK5-induced expression of *Atgl*, we have used adipocytes expressing MK5-S115E (which present increased abundance of ERK3) and wild-type form of MK5, depleted from FOXO1. As expected stimulation of cells with β-agonist or expression of MK5-S115E mutant resulted in induction of *Atgl* expression, which was ameliorated by silencing of FOXO1 (Fig. 4I). Next, we check whether the reintroduction of ATGL to the adipocytes is sufficient to restore the lipolytic rate in cells depleted from ERK3. In fact, overexpression of ATGL in 3T3L1 cells depleted from ERK3 restored the lipolysis rate induced by β-agonist to the level observed in wild-type cells (Fig. 4J). Altogether, these data suggest that ERK3/MK5 drives lipolysis by promoting FOXO1 nuclear translocation and FOXO1-dependent transcription of *Atgl*.

β-Adrenergic stimulation rapidly activates lipolysis. However, transcriptional events that are apparently used by EKR3/MK5 pathway require longer periods in order to be translated into biological effects. Therefore, we have assessed the detailed timing of β-adrenergic activation of ERK3. Already, 15 min after stimulation by β-agonist, ERK3 has been stabilized in adipocytes and the peak of its abundance has been observed from 2 to 8 h later (Supplemental Fig. S4g). Interestingly, β-adrenergic stimulation, induced *Atgl* expression already after 30 min and abundance of the protein was substantially increased 2 h later (Supplemental Fig. S4g,h). Of note, β-adrenergic induced expression and abundance of ATGL, but not the expression of *Mk5*, was ameliorated by the silencing of *Erk3* (Supplemental Fig. S4g–i). Although induction of lipolysis was rapid upon β-agonist, FFAs release kept increasing at later time points in control cells but not in the ERK3-deficient adipocytes (Supplemental Fig. S4j). Therefore, a β-adrenergic-induced ERK3/MK5 pathway is required rather for sustaining lipolysis after the initial phase of the TG release.

### Murine adipose tissue requires ERK3 to perform lipolysis

**Figure 5.**
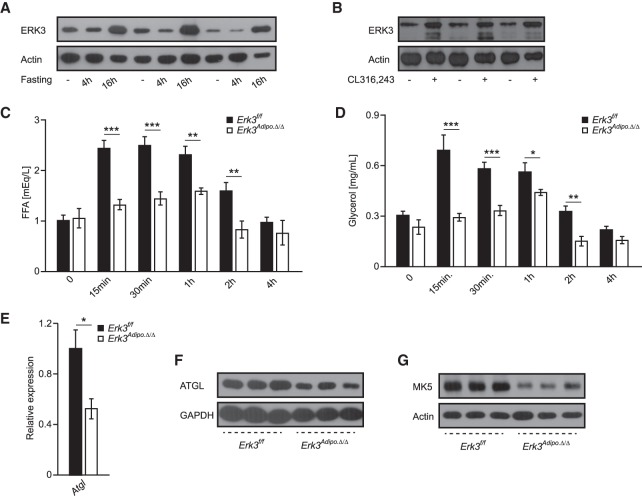
ERK3 is required for induction of lipolysis in adipose tissue of mice. (*A*) ERK3 protein levels in EpiWAT of mice fasted for indicated time points (*n* = 3). (*B*) ERK3 levels in mice injected with β-agonist CL316243 or vehicle control (*n* = 3). (*C*,*D*) FFAs and glycerol release in circulation respectively after injection of β-agonist (CL316243) for indicated times in mice with adipocyte-specific deletion of Erk3 (*Erk3^Adipo.Δ/Δ^*) or control animals (*Erk3f/f*) (*n* = 6). (*E*) Relative Atgl expression in EpiWAT from *Erk3^Adipo.Δ/Δ^* and control mice. (*F*,*G*) ATGL and MK5 levels, respectively, in EpiWAT isolated from *Erk3f/f* and *Erk3^Adipo.Δ/Δ^*. Data are presented as average ± SEM. (*) *P* ≤ 0.05; (**) *P* ≤ 0.01; (***) *P* ≤ 0.001.

Next, we decided to investigate whether our finding of ERK3/MK5 pathway is also relevant in vivo. ERK3 is a constitutively active kinase (Coulombe et al. 2003, 2004) whose abundance is regulated during tissues and tumor development. Consistently, ERK3 has been implicated in lung development and in carcinogenesis (Klinger et al. 2009; Long et al. 2012). However, its role in adipose tissue has not been described. Food deprivation induces stress hormones (catecholamines) and, consequently, lipolytic response in adipose tissue depots, with the strongest response in epi-gonadal white adipose tissue (EpiWAT). Consistently, we found elevated ERK3 levels in EpiWAT of mice subjected to fasting (Fig. 5A). Similarly, β-adrenergic stimulation of mice led to an increase of ERK3 levels in EpiWAT (Fig. 5B), suggesting that ERK3 controls lipolysis rate also under physiological conditions.

To test whether ERK3 controls lipolytic response in adipose tissue, we generated mice deficient for *Erk3* specifically in adipocytes (*Erk3^Adipo.Δ/Δ^*) (Supplemental Fig. S5a,b). Consistently, with the results obtained in vitro β-adrenergic stimulation failed to induce FFAs and glycerol levels in the blood of *Erk3^Adipo.Δ/Δ^* mice, in contrast to control animals (Fig. 5C,D). Thus, ERK3 is required for stimulation-induced lipolysis in adipocytes in animals. Consistent with the mechanism of ERK3 action predicted based on in vitro data, deletion of ERK3 in adipocytes resulted in decreased *Atgl* transcription and protein levels and reduction in MK5 abundance (Fig. 5E–G). Thus, ERK3 is required for lipolysis in adipocytes in vitro and in vivo.

### Deletion of ERK3 in adipocytes promotes energy expenditure

Typically, reduced lipolysis leads to accumulation of fat tissue and the development of obesity and diabetes. Paradoxically, it has been shown that reducing ATGL action can also protect against diabetes (Haemmerle et al. 2006; Arner and Langin 2014; Schoiswohl et al. 2015; Schreiber et al. 2015). In addition to its role regulating *Atgl* expression, FOXO1 promotes differentiation of adipocytes and suppresses expression of uncoupling protein 1 (UCP1), which drives energy expenditure and therefore counteracts lipid accumulation (Nakae et al. 2003, 2008, 2012; Liu et al. 2016; Kita et al. 2019; Peng et al. 2019).

**Figure 6.**
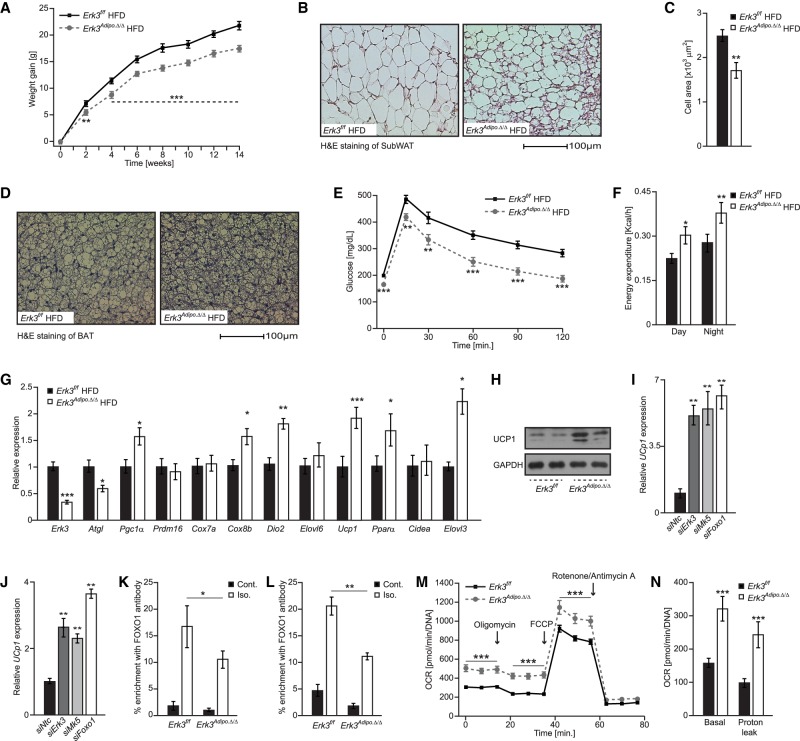
Deletion of Erk3 in adipocytes promotes energy dissipation and protects against obesity. (*A*,*E*) Bodyweight evolution and glucose tolerance test (GTT), respectively, on *Erk3^Adipo.Δ/Δ^* and *Erk3f/f* mice fed a high-fat diet (HFD) (*n* = 11). (*B*,*C*) Representative H&E staining of subcutaneous adipose tissue and quantification of the average adipocyte size of indicated mice fed HFD (*n* = 8). (*D*) Representative H&E staining of brown adipose tissue (BAT) on the indicated mice fed HFD. (*F*) Energy expenditure of *Erk3^Adipo.Δ/Δ^* and *Erk3f/f* mice fed HFD (*n* = 8). (*G*) Relative expression of indicated genes in SubWAT from the indicated mice (*n* = 6). (*H*) Western blot for UCP1 in SubWAT from *Erk3^Adipo.Δ/Δ^* and *Erk3f/f* mice fed HFD (*n* = 6). (*I*,*J*) Relative expression of Ucp1 in subWAT-derived (*I*) and BAT-derived (*J*) adipocytes depleted of specific proteins by siRNA (*n* = 4). (*K*,*L*) Chromatin immunoprecipitation of Ucp1 promoter using FOXO1 antibody from and SVC-derived brown adipocytes (*K*) and white adipocytes (*L*) stimulated for 2 h as shown (*n* = 4). (*M*,*N*) Oxygen consumption rate (OCR) in response to the indicated substances (*M*) as well as OCR annotated to the indicated cellular processes (*N*) in Erk3-deficient SVCs differentiated into brown adipocytes. Data are presented as average ± SEM. (*) *P* ≤ 0.05; (**) *P* ≤ 0.01; (***) *P* ≤ 0.001.

Because of the direct action of ERK3/MK5 complex on the FOXO1-mediated transcriptional signaling, we decided to test the impact of ERK3 on the development of obesity and diabetes in vivo. *Erk3^Adipo.Δ/Δ^* mice gained significantly less weight and presented reduced adiposity than control animals when fed a high-fat diet (HFD) (Fig. 6A; Supplemental Fig. S6a). Moreover, histological analysis revealed reduced adipocytes’ size and multilocular cells within subcutaneous adipose tissue (SubWAT) (Fig. 6B,C) as well as smaller lipid droplets in brown adipose tissue (Fig. 6D). *Erk3^Adipo.Δ/Δ^* mice also displayed improved glucose tolerance and insulin sensitivity (Fig. 6E; Supplemental Fig. S6c) when fed HFD. Interestingly, *Erk3^Adipo.Δ/Δ^* mice fed HFD presented strongly improved fasting glycemia and normalization of glucose during glucose tolerance test to the initial values strongly ameliorated the differences between genotypes (Supplemental Fig. S6b). In the case of insulin challenge, a relative drop in glucose levels compared with values at fasting was similar in *Erk3^Adipo.Δ/Δ^* and *Erk3^f/f^* mice (Supplemental Fig. S6d). On normal diet (ND), *Erk3^Adipo.Δ/Δ^* mice presented similar body weight gain as the corresponding control animals, while glucose clearance and insulin sensitivity were improved only due to the reduced fasting glucose levels (Supplemental Fig. S6e–i). These phenotypes prompted us to test the effect of ERK3 on energy expenditure. Indeed, *Erk3^Adipo.Δ/Δ^* mice fed HFD revealed increased energy expenditure when compared with control animals (Fig. 6F), while energy intake and voluntary movements were unchanged independently of the diet (Supplemental Fig. S6j–m). Elevated expression of *Ucp1* in different adipose tissue depots drives energy dissipation of adipocytes, which can increase the energy expenditure of the entire organism and prevent the development of obesity (Kajimura et al. 2015). In the HFD-fed *Erk3^Adipo.Δ/Δ^* mice, we found increased expression of *Ucp1* and associated genes in SubWAT and brown adipose tissue (BAT) (Fig. 6G; Supplemental Fig. S6n). Consistently, UCP1 protein levels were elevated in SubWAT (Fig. 6H). Moreover, silencing of *Mk5, Erk3* or *FoxO1* was sufficient to promote expression of *Ucp1* and associated thermogenic genes in adipocytes derived from SVC isolated from subWAT (Fig. 6I; Supplemental Fig. S6o) and BAT (Fig 6J; Supplemental Fig. S6p). Moreover, we showed that in response to β-adrenergic stimulation, FOXO1 binds to the promoter of *Ucp1* of white (Fig. 6K) and brown (Fig. 6L) primary adipocytes in ERK3-dependent manner. All of these suggest that ERK3/MK5/FOXO1 axis exerts negative feedback on *Ucp1* expression and presumably energy dissipation by adipocytes. Indeed, isolated primary brown adipocytes from *Erk3^Adipo.Δ/Δ^* mice had an increased oxygen consumption rate and decoupling activity (Fig. 6M,N). Negative energy balance generated by elevated *Ucp1* expression in adipose tissue of mice could be ameliorated by housing animals at thermoneutral conditions (Löffler et al. 2018). In fact, housing HFD fed *Erk3^Adipo.Δ/Δ^* mice and corresponding control animals at thermoneutral conditions resulted in similar body weight gain (Supplemental Fig. S6q). This suggests that *Erk3^Adipo.Δ/Δ^* mice are protected from HFD-induced obesity due to the increased thermogenesis.

Thus, depletion of ERK3 inhibits lipolysis by reducing FOXO1-mediated ATGL expression but at the same time promoting *Ucp1* expression and energy dissipation by brown as well as white adipose tissue, thereby protecting against obesity and diabetes. By promoting FOXO1 nuclear localization, ERK3 may fine-tune the transcriptional response leading to stimulation of *Atgl* and reduction of *Ucp1* expression. Thus, at least in the context of HFD, FOXO1-dependent suppression of *Ucp1* expression and energy expenditure appears to exert a dominant role over ATGL-dependent lipolysis. Thus, *Erk3^Adipo.Δ/Δ^* mice provide us with the unique experimental model to test the physiological relevance of both transcriptional responses.

### Inactivation of ERK3 in obese mice prevents further body weight gain and improves insulin sensitivity

**Figure 7.**
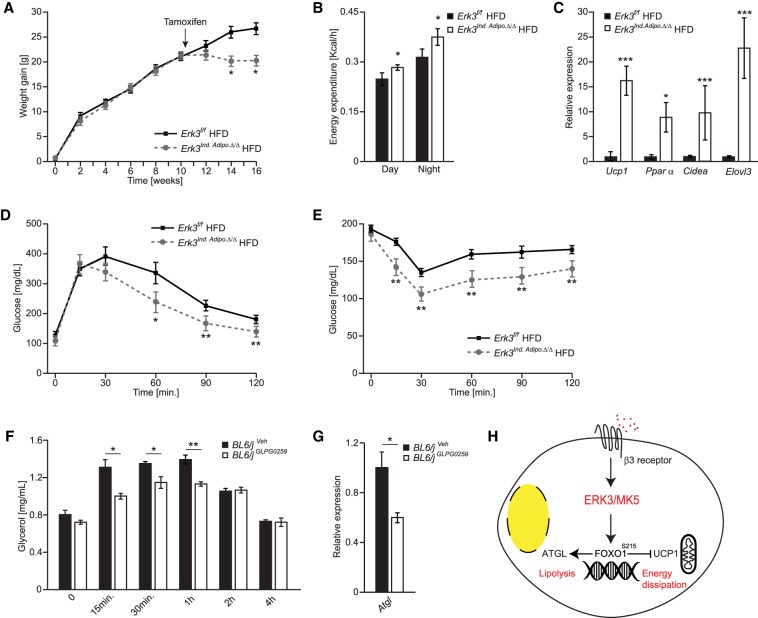
Inhibiting ERK3/MK5 pathway in obese mice promotes energy dissipation and prevents further body weight gain. (*A*–*E*) Bodyweight evolution (*A*), energy expenditure (*B*), expression of thermogenic genes (*C*), glucose tolerance test (*D*), and insulin tolerance test (*E*) of mice with inducible deletion of Erk3 (*Erk3^Ind.Adipo.Δ/Δ^*) and control animals fed HFD upon administration of tamoxifen at indicated time point. (*F*) Glycerol release in circulation after injection of β-agonist (CL316243) for indicated times in BL6/J mice that have been orally administered with GLPG0259 inhibitor (10 mg/kg) or vehicle control for one consecutive week (*n* = 5). (*G*) Relative Atgl expression in SubWAT from GLPG0259 administered mice and their respective vehicle control mice (*n* = 5). (*H*) Proposed model of action of the β-adrenergic-induced ERK3/MK5/FOXO1 pathway. Data are presented as average ± SEM. (**) *P* ≤ 0.005; (***) *P* ≤ 0.01.

To corroborate this hypothesis, we tested whether deletion of ERK3 in mice with established obesity can inhibit further body weight gain and improve glycaemia. We have crossed ERK3^f/f^ with tamoxifen-inducible Adiponectin promoter-driven CRE line (*Erk3^Ind.Adipo.Δ/Δ^*) and induced deletion of ERK3 with tamoxifen administration after 9 wk of HFD feeding. After induction of ERK3 deletion in *Erk3^Ind.Adipo.Δ/Δ^* (Supplemental Fig. S7a), these mice did not further gain weight like corresponding control animals (Fig. 7A). Consistent with previous results, *Erk3^Ind.Adipo.Δ/Δ^* mice displayed elevated energy expenditure (Fig. 7B), but deletion of ERK3 did not influence food intake and voluntary movements (Supplemental Fig. S7b,c). As in the case of constitutive deletion of ERK3 in the adipocytes, induction of ERK3 removal in mice with established obesity resulted in elevated expression of *Ucp1* and other thermogenic genes in subWAT and BAT (Fig. 7C; Supplemental Fig. S7d,e). Remarkably, induced deletion of ERK3 in obese mice improved glucose tolerance and insulin sensitivity (Fig. 7D,E). Therefore, inhibition of ERK3/MK5 pathway might ameliorate obesity-induced diabetes. In fact, MK5 inhibitor (GLPG0259) has been already developed and approved for phase II clinical trial (Westhovens et al. 2013). One-week administration of GLPG0259 in mice significantly reduced β-agonist-induced FFAs and glycerol levels as well as *Atgl* expression compared with control animals (Fig. 7F,G). Finally, inhibition of MK5 resulted in the induction of thermogenic genes expression in subWAT and BAT (Supplemental Fig. S7f,g). Altogether, these indicate that inhibition of MK5 with GLPG0259 might be a valid strategy to target ERK3/MK5/FOXO1 pathway in adipocytes.

Taken together, we identified a signaling axis that can link and fine-tune the transcriptional response leading to lipolysis and to energy dissipation in adipocytes. We speculate that inhibition of ERK3/MK5 signaling, which could be achieved by specific inhibitors, might represent an attractive strategy to ameliorate diabetes and obesity (Fig. 7H).

## Discussion

Aberrant activation of lipolytic machinery in adipocytes contributes to the development of obesity-evoked diabetes (Haemmerle et al. 2006; Arner and Langin 2014; Schoiswohl et al. 2015; Schreiber et al. 2015). Ablation of the major enzyme involved in the regulation of lipolysis, ATGL, in adipose tissue and in whole-body results in the increase of TG accumulation in adipocytes and improved insulin sensitivity (Trites and Clugston 2019). Paradoxically, pharmacological inhibition of major lipase ATGL by atglistatin has been shown to protect mice from the development of obesity and diabetes (Schweiger et al. 2017). However, atglistatin is not effective in humans (Schweiger et al. 2017). Therefore, there is an urgent need for identification of targets, inhibition of which would decrease the lipolysis rate and could potentially protect from the development of obesity-evoked diabetes. Kinases can be in general easily targeted by pharmacological agents. Here, we developed a screening strategy to identify kinases implicated in the regulation of lipolysis rate. Our primary and secondary screen revealed 50 kinases implicated in the regulation of lipolysis induced by adrenergic and serotonergic stimulation. Among them, several kinases were previously implicated in the regulation of lipolysis or adipocyte function. For example, our screen revealed that silencing of two regulatory subunits of multisubunit complex of PKA (*Prkar1a* and *Prkar2b*, which inhibits PKA activation) (Torres-Quesada et al. 2017) enhances the lipolysis rate. On the other hand, silencing of *Ndrg1*, *Raf1*, *Lats2*, *Peg3*, and *Trib3*, which were previously shown to promote lipolysis or to regulate other aspects of adipocytes function (Curley et al. 2005; Qi et al. 2006; Kalderon et al. 2012; An et al. 2013; Cai et al. 2017), resulted in inhibition of lipolysis. These results confirm the appropriate design and relevance of our screen. Among the known factors not identified in the screen were the classical members of MAPK family, extracellular-regulated kinase (ERK) 1/2 and c-Jun N-terminal kinase (JNK) 1/2/3 reported previously to promote triglycerides degradation in adipocytes (Greenberg et al. 2001; Gehart et al. 2010; Hong et al. 2018). This might be explained by the fact that in our screen we silenced each of the isoforms of a given family of kinases separately. Since members of the MAPK family presents high homology, high redundancy in function can occur between different isoforms. Alternatively, in our screen, we stimulated lipolysis with a mixture of β-adrenergic and HTR2B agonists, which might not necessarily lead to the activation of classical members of the MAPK family. Excitingly, the screen revealed that atypical member of MAPK family, ERK3, is a critical regulator of lipolysis. We showed that deletion of ERK3 in adipose tissue of mice suppressed the production of FFAs and glycerol in response to the β-adrenergic stimulation, ultimately confirming results from the screen.

ERK3 is a constitutively active kinase (Coulombe et al. 2003, 2004) whose protein levels are tightly regulated. We show that in adipocytes ERK3 activity correlates with its abundance. Moreover, we propose a mechanism whereby the formation of a complex between ERK3 and MK5 kinase depends on β-adrenergic-induced PKA activation and promotes the stability of both kinases. In line with our model, β-adrenergic stimulation promotes interaction between MK5 and ERK3. Similarly, the phosphomimetic mutant of MK5 (MK5-S115E) presented a higher affinity to endogenous ERK3 than the wild-type form of MK5 in the unstimulated cells. However, the expression of phosphomimetic mutant of MK5 also induced stability of ERK3, therefore, theoretically enhanced interaction between MK5 and ERK3 might be a result of increased availability of these proteins. These results provide the first link between adrenergic signaling and atypical MAPKs and define ERK3/MK5 as crucial components of adrenergic-induced signaling. Moreover, our data confirm published evidence that ERK3 is subjected to ubiquitin–proteasome-mediated degradation (Coulombe et al. 2003, 2004). In future, it will be important to understand the molecular mechanisms by which the formation of a complex between ERK3 and MK5 protects both kinases from proteasome-mediated proteolysis.

Our results demonstrate that β-adrenergic-induced ERK3/MK5 pathway promotes lipolysis by driving the expression of major lipase, ATGL, in a FOXO1-dependent manner. These data explain how β-adrenergic signaling is linked to the transcription of lipolytic enzymes in particular FOXO1-dependent transcription. At this point, we cannot exclude the possibility that ERK3/MK5 pathway may, in addition, promote lipolysis in a transcriptional-independent manner. Also, we cannot exclude that ERK3 partially drives lipolysis and thermogenesis in an MK5 independent manner. In fact, the silencing of *Mk5* was less effective in suppressing lipolysis than the silencing of *Erk3*. However, these discrepancies between phenotypes might also reflect differences in siRNA efficiency.

We showed that the ERK3/MK5 pathway drives lipolysis by promoting *Atgl* expression. β-Adrenergic stimulation induces lipolytic machinery within minutes, while transcriptional response requires at least 1 h in order to translate into a biological effect. In fact, the silencing of ERK3 resulted in a significant decrease in lipolysis rate only 2 h after β-adrenergic stimulation, indicating that the ERK3/MK5 pathway is responsible for sustaining lipolysis rather than initializing it. However, we cannot exclude that the deletion of *Erk3* leads to the reduction of *Atgl* expression prior to the β-adrenergic stimulation.

FOXO1 regulates a broad spectrum of transcriptional targets implicated in different aspects of adipocytes function. We and others showed that FOXO1 promotes the expression of *Atgl* (Chakrabarti and Kandror 2009b; Chakrabarti et al. 2011; Jung et al. 2019). Interestingly, a number of reports showed that FOXO1 suppresses the expression of genes regulating energy dissipation, including UCP1, in white and brown adipocytes (Nakae et al. 2008, 2012; Liu et al. 2016; Kita et al. 2019; Peng et al. 2019). However, a recent study indicated that in brown adipose tissue of mice fed ND, FOXO1 does not affect UCP1 and in vitro under specific culture conditions deletion of FOXO1 might even decrease *Ucp1* expression in brown adipocytes (Jung et al. 2019). Another study also indicates that in the mice with decreased insulin signaling, FOXO1 might drive *Ucp1* expression (Ortega-Molina et al. 2012). We showed that expression of *Ucp1* was markedly induced in white and, even more relevant to the total energy balance of animals also, in brown adipocytes deficient for FOXO1, MK5, or ERK3. Consistent with these observations, we showed that deletion of ERK3 in adipocytes of mice inhibits the development of obesity or further weight gain in obese mice, at least partially due to the increased *Ucp1* expression and increased energy dissipation.

Several reports indicate that the induction of lipolysis is required for UCP1-dependent energy dissipation by adipocytes (Ahmadian et al. 2011; Li et al. 2014; Schreiber et al. 2017). Deletion of ATGL in the whole body or all of the depots of adipose tissue results in decreased energy dissipation in mice upon cold exposure especially during the time of food deprivation (Ahmadian et al. 2011; Schreiber et al. 2017). However, in mice deficient for ATGL in the whole body (except for the cardiac muscle) and mice treated with atglistatin, which were fed HFD and maintained at room temperature, energy expenditure was not decreased (Schreiber et al. 2015, 2017). Moreover, mice treated with Atglistatin display higher UCP1 levels in adipose tissue (Schweiger et al. 2017). Similarly, deletion of CGI-58 in brown adipose tissue, which partially blocks lipolysis, does not block energy dissipation by adipocytes in mice fed ad libitum (Shin et al. 2017, 2018). Mice deficient for ERK3 show about 50% reduced *Atgl* expression, and at the same time, increased *Ucp1* expression in white as well as in brown adipose tissue when fed HFD. Moreover, these mice show increased energy expenditure when fed HFD and maintained under room temperature. These results are in line with the fact that FOXO1 promotes *Atgl* expression but suppresses *Ucp1* transcription (Nakae et al. 2008, 2012; Chakrabarti and Kandror 2009b; Chakrabarti et al. 2011; Liu et al. 2016; Kita et al. 2019; Peng et al. 2019). Therefore, it appears that at least upon HFD feeding, suppression of *Ucp1* expression and energy expenditure would play a dominant role over ATGL-dependent lipolysis. We postulate that increased expression of *Ucp1* in mice deficient for ERK3 in adipocytes fed ad libitum with HFD at least partially contributes to the enhanced energy expenditure observed in these animals, which in turn protects from HFD-induced obesity. However, at this point we cannot exclude additional mechanisms promoting energy expenditure in the absence of ERK3.

Based on our results, pharmacological targeting of the ERK3/MK5/FOXO1 pathway might be a valid strategy to treat obesity and associated diabetes. Moreover, the existence of MK5 inhibitor, GLPG0259, which was already shown not to have toxic side effects in humans (Westhovens et al. 2013), raises a possibility for effective antiobesity therapy. In fact, we have shown that administration of GLPG0259 in mice reduces *Atgl* expression, suppresses lipolysis in mice, and increases the expression of thermogenic genes, indicating that this inhibitor effectively targets ERK3/MK5/FOXO1 pathway. However, since the deletion of *Atgl* had a deleterious effect on heart function and currently available inhibitors against ATGL are not effective in humans (Trites and Clugston 2019), targeting directly ATGL for treatment of obesity and diabetes is difficult. Targeting ERK3/MK5 not only partially inhibits lipolysis but also promotes energy dissipation; thus, inhibitors targeting this pathway might be of use for the treatment of obesity and associated diseases. However, further studies are required to define whether inhibitors directed against the ERK3/MK5 pathway might overcome the potential side effects of ATGL inhibitors.

Taken together, we identified the β-adrenergic-induced ERK3/MK5/FOXO1 pathway as a central regulator of lipolysis and energy expenditure and is a potential target for future antiobesity therapy (Fig. 7G).

## Materials and methods

### Preadipocyte culture and differentiation

3T3-L1 preadipocytes, Platinum-E, and HEK293T cells were cultured and maintained in Dulbecco's modified Eagle's medium (DMEM), 10% fetal bovine serum (FBS), 1% nonessential amino acids (NEAA), and 40 µg/mL gentamycin. Stromal vascular cells (SVCs) were isolated from subcutaneous white adipose tissue (sWAT) or from brown adipose tissue (BAT) of 5- to 9-wk-old mice as described previously in Cai et al. (2017). Concisely, mice subcutaneous white adipose tissue (SubWAT) and brown adipose tissue (BAT) were sliced and digested in PBS containing 2 mg/mL collagenase D (Sigma-Aldrich), 5 mM CaCl_2_, and 1% BSA for 40 min at 37°C. Adipose tissue are then passed through a 40-µm mesh, washed in PBS by centrifugation, and plated on Matrigel-coated (Corning) plates in DMEM/F-12 containing 10% FBS, 1% NEAA, and 40 µg/mL gentamycin. Two days after confluence, preadipocyte differentiation was induced by 0.2 µM indomethacin, 0.5 mM IBMX, 1 µM dexamethasone, and 1.5 µg/mL insulin for the first 48 h. Afterward, cells were maintained in complete medium with 1.5 µg/mL insulin for up to six additional days. In preadipocytes derived from BAT, additional T3 (2 nM; Sigma-Aldrich) was added to the medium throughout differentiation. 3T3-L1 cells were differentiated into adipocytes according to the standard procedure described in Cai et al. (2017). Prior to treatment, differentiated adipocytes were washed twice with phenol-free DMEM and then serum-starved for 2 hr in phenol red-free DMEM supplemented with 2% BSA; afterward and as indicated in experiments, treatments were added in the following concentrations: 1.10 µM isoproterenol (Tocris Biotech), 10 µM Mg-132 (Tocris Biotech), 20 μM Forskolin (Sigma-Aldrich),0.5 mM IBMX (Sigma-Aldrich), 10 μM CL316,243 (Tocris Biotech), 20 µM lactacystin (Sigma-Aldrich), 100 μg/mL cycloheximide (Tocris Biotech), 20 μM H89 (Sigma-Aldrich), 20 μM PKI14-22 (Sigma-Aldrich), 50 nM leptomycin B1 (Sigma-Aldrich), and 10 μM BW-723C86 (Tocris Biotech).

### Transient transfection with siRNA

Differentiated adipocytes were transfected with siRNA always in suspension as follows: Mature adipocytes were detached with Accutase solution for 10 min, spun down at 300*g* for 5 min, and counted. Indicated siRNAs and Dharmafect-Duo transfection reagent (Dharmacon) were diluted in Opti-MEM I reduced serum medium separately before being mixed by pipetting. The siRNA-Duofect mix was added to Matrigel-coated culture plates or 96-well Seahorse plates and left to incubate for 30 min at room temperature. The cells were resuspended in culture medium and added on top of the preincubated siRNA-Duofect mix. The final concentrations of Duofect, siRNA, and cell number were adjusted per surface area in a ratio of 2.1 uL/cm^2^ duofect, 1.25 nM/cm^2^ siRNA, and 16.4 × 10^4^/cm^2^ cells, respectively. The cells were used for experiments 48 h after transfection. All siRNA sequences were purchased as smart pools from Dharmacon.

### Generation of stable cell lines

The Dharmacon GIPZ lentiviral shRNA system was used to induce long-term gene silencing of the *Erk3* gene, shRNA *Erk3* oligo was synthesized by Eurofins (sh*Erk3*: 5′-TGCTGTTGACAGTGAGCGAACACCTGTAACTACAAAACAATAGTGAAGCCACAGATGTATTGTTTTGTAGTTACAGGTGTGTGCCTACTGCCTCGGA-3′) and cloned into a pGIPZ shRNA mir (a generous gift from Eilers laboratory, Biocenter, Würzburg) between XhoI and EcoRI cloning sites. The pBABE- Puro retroviral vector system was used to overexpress ERK3 protein. Concisely, Myc tagged *Erk3* coding sequence was cloned from human cDNA vector and inserted between EcoR1 and Sal1 in the pBABE vector. HA-Foxo1 and 3xFlag-Mk5, Myc-mPnpla2 overexpressing vectors were all synthesized by Vectorbuilder by the insertion of their respective coding sequences in PLV-Puro lentivirus plasmid. Wild-type sequences were point mutated as mentioned above using Q5 site-directed mutagenesis kit (NEB). Lentiviral particles were packaged in HEK293T cells with psPAX and pMD2.G and transduced in 3T3L1 cells. Retroviral particles were produced using PlatinumE cells. 3T3L1 cells were spinfected with viral supernatant with the addition of polybrene then selected by puromycin 48 h later.

### Lipolysis assay

Determination of free glycerol and free fatty acids was performed as described before (Löffler et al. 2018). In brief, adipocytes were washed twice with phenol-free DMEM then serum-starved for 2 h in phenol red-free DMEM supplemented with 2% fatty acid-free BSA, stimulated afterward with 1 µM isoproterenol for 2 h. Conditioned medium was then collected and analyzed. FFAs in the medium were measured using NEFA reagents (Wako) and glycerol was quantified by free glycerol reagent (Sigma-Aldrich) according to the manufacturers’ instructions. Values were normalized to the cells’ total DNA content by Hoechst 33342 staining.

### Oxygen consumption

Mitochondrial respiration of brown adipocytes derived from the stromal-vascular fraction was determined by measuring oxygen consumption rate (OCR) using the Seahorse XF Cell Mito stress test (Agilent Technologies 103015-100) in a Seahorse XFe96 analyzer according to the manufacturer's protocol. Briefly, cells were incubated for 1 h with 180 µL Seahorse assay medium containing 1 mM sodium pyruvate, 2 mM glutamine, and 5 mM glucose. Subsequently, the cells were stimulated with 2 µM oligomycin, 1 µM FCCP, and 0.75 µM rotenone/antimycin A (Sigma-Aldrich). Cells are then fixed and the total DNA content was assessed by crystal violet staining. Absorbance was read at 595 in a Spark 10M microplate reader (Tecan) and the measurements were used to normalize the seahorse values.

### Generation of mouse models

All experiments with mouse models were approved by the local institutional animal care (Regierung von Unterfranken, Germany) and conducted according to the guidelines and state regulations. Experiments were performed under animal protocol numbers AK 55.2-2531.01-124/13 and 55.2-2532-2-741. Mice were maintained in a specific pathogen-free facility with the ambient temperature set at either 23°C or 30°C (for thermoneutrality experiment), following a 12-h light–dark cycle and given ad libitum access to water and standard chow diet (sniff Spezialdiäten), which was exchanged under indicated experimental conditions to high-fat diet (HFD). All mice were closely monitored by the authors, facility technicians, and by an independent veterinary scientist responsible for animal welfare. Bl6/j mice were administered orally by gavage with either vehicle or 10 mg/kg MK5 inhibitor (GLPG0259; Medkoo 15986) for one consecutive week.

*Erk3 floxed* mice (Erk3^fl/fl^) were genetically engineered on the C57BL/6 background. Briefly, the targeting vector containing part of the *Erk3* gene was constructed as follow: The selection neomycin (Neo)-containing cassette flanked by two FLP sites was inserted between Exons (E) 3 and 4 together with LoxP site. Second LoxP site was inserted between E2 and E3. At the 3′-end of the construct negative selection cassette containing diphtheria toxin was inserted (Supplemental Fig. S3c). The resulting construct was linearized and electroporated into the BL6-derived ES cells. Upon selection of positive clones and expansion of ES cells in which homologs recombination took place, the ES cells were injected into the blastocyst and implanted into a pseudo-pregnant female. The resulting chimeric mice were crossed with the FLPe knock-in mice to remove the Neo cassette. The resulting *Erk3* flox mice (*Erk3^f/f^*) were used for the generation of targeted deletion in tissues. For targeted deletion of *Erk3* in adipocytes, *Erk3^f/f^* mice were cross-bred with adiponectin promoter-driven Cre mice adiponectin-Cre. As for the inducible model, *Erk3^f/f^* mice were cross-bred with adiponectin promoter-driven Cre-ERT2 mice 100 mg/kg tamoxifen (Sigma-Aldrich) was then administered orally when indicated in experiments above for five consecutive days to initiate deletion *Erk3*.

### Animal experiments

For induction of lipolysis, we injected *Erk3^Adipo.Δ/Δ^* and their corresponding control *Erk3^f/f^* mice, BL6/j mice either treated with vehicle or MK5 inhibitor (GLPG0259) as indicated above, with β-adrenergic agonist CL316,243 (CL) intraperitoneally (i.p.) at a concentration of 2 mg/kg in 0.9% NaCl. Blood was sampled from the tail vein for metabolic studies as indicated on the corresponding figures.

Bodyweight gain of mice was monitored every other week. Indirect calorimetry measurements were done at the end using the Phenomaster system (TSE) as described before (Viera et al. 2016; Mayer et al. 2019). Briefly, mice were housed individually with ad libitum access to indicated food and water. After a 48-h acclimation period, parameters were sampled for 72 h in the fed stage. Locomotor activity was determined by consecutive photobeam breaks occurring in adjacent beams. All measurements were obtained every 10 min during a full light/dark cycle.

For blood glucose tolerance tests, mice were fasted overnight, and glucose (2 g/kg) was administered intraperitoneally. For insulin sensitivity tests, mice were fasted for 4 h before receiving an intraperitoneal injection of 0.5 U/kg human insulin. A blood drop was collected from the tail tip directly onto the test strip for the blood glucose measurement (Accu-Chek, Roche). Glucose concentrations were sampled in both conditions before and at 15, 30, 60, 90, and 120 min after injection.

### Histological analysis and cell size analysis

For histological analysis, adipose tissues were fixed in 4% paraformaldehyde and embedded in paraffin. The sections (5 µm) were stained with hematoxylin–eosin (H&E) according to the standard procedure. Digital images were captured using a Leica light microscope at 20× magnification. Measurement of adipocytes size and distribution was performed in a blinded fashion. Approximately 400 adipocytes per sample were measured using ImageJ software with the additional Adiposoft plugin.

### Western blotting and immunoprecipitation and subcellular fractionations

Total proteins were extracted from cells and tissues by a standard RIPA buffer. Subcellular fractionations were performed using the NE-PER kit (Thermo Scientific) according to the manufacturer's instructions. For immunoprecipitation (IP), indicated cells were lysed by Pierce IP lysis buffer (Thermo Scientific) and then IP using Flag tag magnetic beads (Millipore M8823) according to the manufacturer's instructions. The lysis and wash buffers were all supplemented with protease and phosphatase inhibitors mix. Protein concentration was quantified using a BCA kit (Thermo Scientific). Reduced protein extracts were separated on 10% SDS-PAGE gels by electrophoresis and transferred PVDF membranes with wet transfer cells. Membranes were blocked in 5% (w/v) skim milk in TBS-T before overnight probing with the indicated primary antibodies at 4°C, followed by TBST washes and incubation with the corresponding secondary antibody. The signals were detected on autoradiography film with enhanced chemiluminescence solution. Antibodies used included rabbit anti-GAPDH (clone 14C10; Cell Signaling Technology), rabbit anti-Erk3 (clone EP1720Y; Abcam), rabbit anti-MAPKAPK-5 (clone D70A10; Cell Signaling Technology), rabbit anti-FoxO1 (clone C29H4; Cell Signaling Technology), rabbit anti-Atgl (Cell Signaling Technology 2138), rabbit anti-HSL (Cell Signaling Technology 4107), rabbit anti-Phospho HSL Ser606 (Cell Signaling Technology 4126), rabbit anti-PPARγ (clone C26H12; Cell Signaling Technology), rabbit anti-Ucp1 (clone D9D6X; Cell Signaling Technology), rabbit anti-β3 Tubulin (clone D71G9; Cell Signaling Technology), goat anti-Lamin b1 (clone C-20; Santa Cruz Biotechnology), rabbit anti-GAPDH (Sigma-Aldrich G9545), mouse anti-β-Actin (Sigma-Aldrich A5441), and rabbit anti-H3 (Abcam ab1791).

### Immunofluorescence

Indicated cells were seeded onto poly-L-lysine-treated glass coverslips in 12-well culture dishes and treated accordingly. Cells were washed in PBS before fixation with 4% paraformaldehyde for 10 min at room temperature. Cells were permeabilized with 0.5% Triton X-100 in PBS for 15 min at room temperature. To reduce nonspecific background noise, cell were blocked with PBS 5% (v/v) normal goat serum, 0.1% (v/v) Triton X-100, and 0.05% (v/v) Tween 20. FOXO1A (Abcam ab52857) primary antibody incubation was done for 16 h in a humidified chamber at 4°C. After thorough washes, cells were incubated with Alexa Fluor 488 secondary antibody (Life Technologies) and Phalloidin–Atto 647N (Sigma-Aldrich) for 1 h in the dark. Cells were then washed before being mounted onto glass slides using ProLong Gold mounting reagent that contained the nuclear stain 4′,6-diamidino-2-phenylindole (DAPI). Slides were visualized using Leica TCS SP8 confocal microscope.

### Real-time PCR analysis

Total RNA was extracted from cells and tissues using the RNeasy kit according to the manufacturer's protocol. Reverse transcription (RT) was performed using 1 µg of RNA and first strand cDNA synthesis kit (Thermo-Scientific). Quantitative PCR was performed using 10 ng of cDNA, power-up SYBR Green and the respective pair of primer sequences. The genes and sequences used were as follows: *Erk1* (forward: 5′-TCCGCCATGAGAATGTTATAGGC-3′; reverse: 5′-GGTGGTGTTGATAAGCAGATTGG-3′), *Erk2* (forward: 5′-TCAGATGAATTTTCGTTGGCAGA-3′; reverse: 5′-GAGCACTTGGGTTACTCCACG-3′), *Erk3* (forward: 5′-CACATGAACTTGAACAGATGCAG-3′; reverse: 5′-CAGTGCTTCTCGGCTGATCC-3′), *Mk5* (forward: 5′-CCTCATTTCTAGCTTTTGGACGA-3′; reverse: 5′-CTGGGAGCCGGAATTAGTGG-3′), *FoxO1* (forward: 5′-CCCAGGCCGGAGTTTAACC-3′; reverse: 5′-GTTGCTCATAAAGTCGGTGCT-3′), *Atgl* (forward: 5′-CAACGCCACTCACATCTACGG-3′; reverse: 5′-GGACACCTCAATAATGTTGGCAC-3′), *Prdm16* (forward: 5′-ACGGACAGGGCTTCTCCTAC-3′; reverse: 5′-ATGGTGGTATCGAGGGTGGAA-3′), *Pgc-1α* (forward: 5′-AGCGCCGTGTGATTTACGTT-3′; reverse: 5′-CCGCAGATTTACGGTGCATT-3′), *Pparγ* (forward: 5′-GGAAGACCACTCGCATTCCTT-3′; reverse: 5′-GTAATCAGCAACCATTGGGTCA-3′), *Pparα* (forward: 5′-AACATCGAGTGTCGAATATGTGG-3′; reverse: 5′-CCGAATAGTTCGCCGAAAGAA-3′), *Ucp1* (forward: 5′-AGGCTTCCAGTACCATTAGGT-3′; reverse: 5′-CTGAGTGAGGCAAAGCTGATTT-3′), *Rpl13a* (forward: 5′-CCCTCCACCCTATGACAAGA-3′; reverse: 5′-GCCCCAGGTAAGCAAACTT-3′), *Dio2* (forward: 5′-TGTCTGGAACAGCTTCCTCC-3′; reverse: 5′-CCATCAGCGGTCTTCTCCG-3′), *Cedia* (forward: 5′-TGACATTCATGGGATTGCAGAC-3′; reverse: 5′-GGCCAGTTGTGATGACTAAGAC-3′), *Cox7a* (forward: 5′-AGGACGCAAAATGAGGGC-3′; reverse: 5′-TCTTGTGGGGGAAGGAGG-3′), *Cox8b* (forward: 5′-TGTGGGGATCTCAGCCATAGT-3′; reverse: 5′-AGTGGGCTAAGACCCATCCTG-3′), *Tmem26* (forward: 5′-ACCCTGTCATCCCACAGAG-3′; reverse: 5′-TGTTTGGTGGAGTCCTAAGGTC-3′), *Elovl3* (forward: 5′-TTCTCACGCGGGTTAAAAATGG-3′; reverse: 5′-GAGCAACAGATAGACGACCAC-3′), *Elovl6* (forward: 5′-CCCGAACTAGGTGACACGAT-3′; reverse: 5′-CCAGCGACCATGTCTTTGTA-3′), *Cited 1* (forward: 5′-AACCTTGGAGTGAAGGATCGC-3′; reverse: 5′-GTAGGAGAGCCTATTGGAGATGT-3′), and *Tnfrst9* (forward: 5′-CGTGCAGAACTCCTGTGATAAC-3′; reverse: 5′-GTCCACCTATGCTGGAGAAGG-3′).

### RNA sequencing

RNA sequencing was performed as described previously (Löffler et al. 2018). Briefly, total RNA was extracted from differentiated 3T3-L1 cells transfected with siRNA targeting E*rk3* or a nontargeting sequence. Afterwards, mRNA was isolated using the NEBNext poly(A) mRNA magnetic isolation module (New England Biolabs) and library preparation was performed with the NEBNext Ultra RNA library preparation kit for Illumina following the instruction manual. Libraries were size-selected using Agencourt AMPure XP beads (Beckman Coulter) followed by amplification with 12 PCR cycles. Library quantification and size determination were performed with the fragment analyzer system (Thermo Fisher Scientific, Agilent). The libraries were sequenced with an Illumina genome analyzer IIx or an Illumina NextSeq 500. FASTQ files were generated using the bcl2fastq Conversion Software v2.19. Differential gene expression was calculated with the R-based software tool edgeR. The RNA sequencing raw and processed data sets, as well as information on data processing, are available at accession number GSE142424

### Chromatin immunoprecipitation (ChIP) assay

ChIP studies were carried out using the ChIP kit (Abcam) according to the manufacturer's instructions. Briefly, after crosslinking, nuclei extracted from indicated 3T3-L1 mature adipocytes were lysed and sonicated for 15 (on/off) cycles of 15 sec each. FOXO1 proteins were then immunoprecipitated from precleared lysates using chip-grade FOXO1 antibody or Actin antibody as a negative control. Protein–DNA complexes were eluted and treated with proteinase K. Purified DNA was analyzed by quantitative PCR using SYBR Green reaction with the following primers: 5′-AGGTTTCTAGTTTAGGATTGAAG-3′ and 5′-ACCTGAGCAGTAGTTATATACAT-3′ (–1041 to –1158 nt of the *Atgl* promoter) as described in Chakrabarti and Kandror (2009a) and the *Ucp1* promoter primers (5′-CTGTTGTTGCTGCTGCTGTT-3′ and 5′-GGAAGCTGCAAGACCTATGG-3′) as described in Ortega-Molina et al. (2012). The percentage of enrichment was calculated based on percent input DNA after adjusting for total input DNA: 100 × 2^(adjusted input Ct − IP Ct)^.

## Supplementary Material

Supplemental Material
