# Supplementary material for: The adrenergic-induced ERK3 pathway drives lipolysis and suppresses energy dissipation
Source: Genes Dev. 2020 Apr 1;34(7-8):495–510. doi: 10.1101/gad.333617.119 (PMC7111262; doi:10.1101/gad.333617.119)
Supplement: Supplemental Material [file supp_34_7-8_495__index.html]

The adrenergic-induced ERK3 pathway drives lipolysis and suppresses energy dissipation — Supplemental Material 

# The adrenergic-induced ERK3 pathway drives lipolysis and suppresses energy dissipation

## Supplemental Material

- Supplemental\_Data.pdf
- Supplemental\_Table\_S1.xlsx
