# Supplementary material for: The adrenergic-induced ERK3 pathway drives lipolysis and suppresses energy dissipation
Source: Genes Dev. 2020 Apr 1;34(7-8):495–510. doi: 10.1101/gad.333617.119 (PMC7111262; doi:10.1101/gad.333617.119)
Supplement: Supplemental Material [file supp_gad.333617.119_Supplemental_Data.pdf]

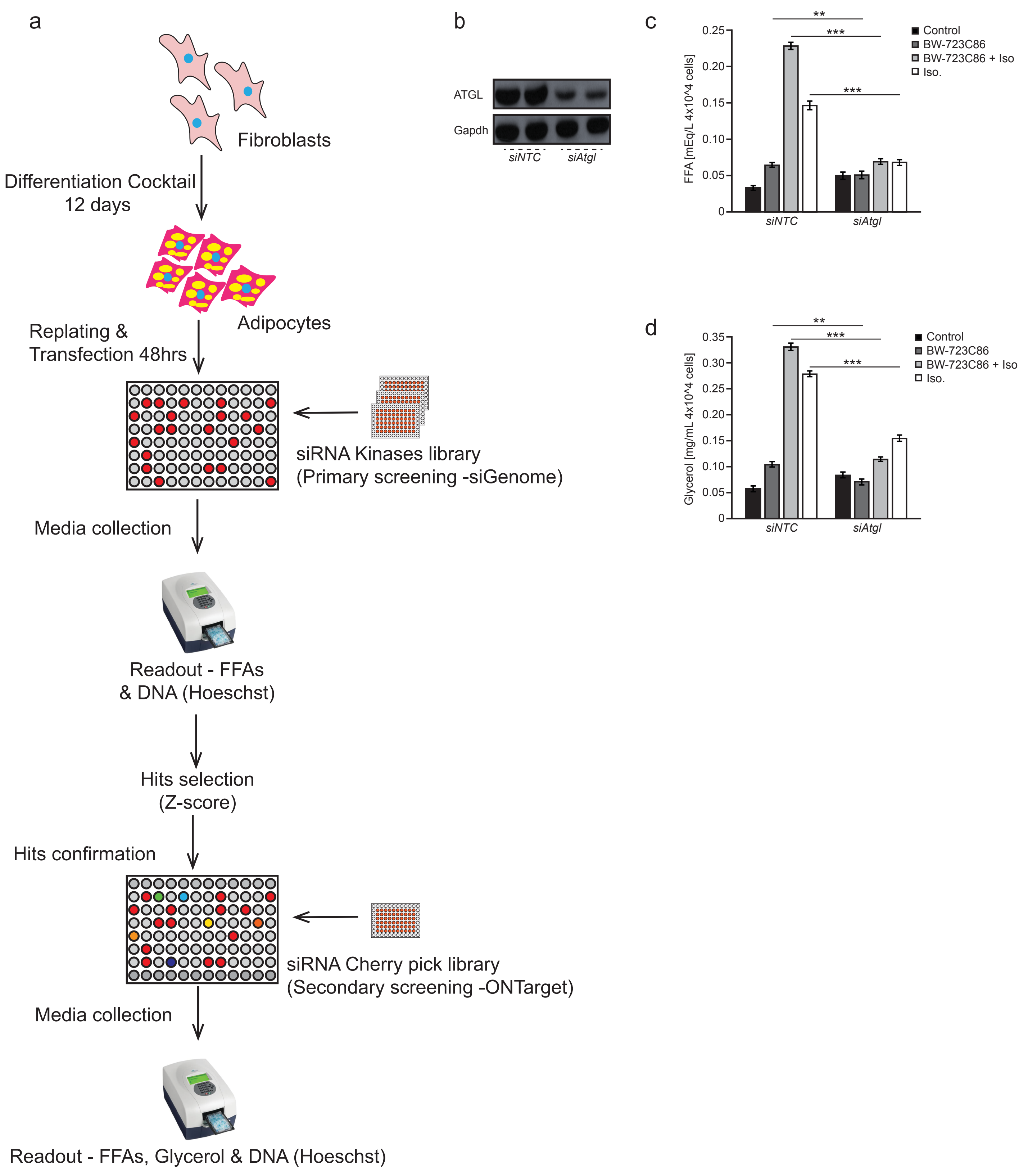

Supplemental Fig. S1 Design of the screen for kinases regulating lipolysis. (a) Schematic representation of the screening strategy. (b) Western blot (WB) analysis of differentiated 3T3L1 cells transfected with siRNA against *Atgl*. Free fatty acids (FFAs) (c) and glycerol (d) output from 3T3L1 adipocytes transfected and stimulated as indicated. n=3 for each experiment, for graphs - data presented as average +/- SEM, ** P ≤0.01, *** P ≤ 0.001

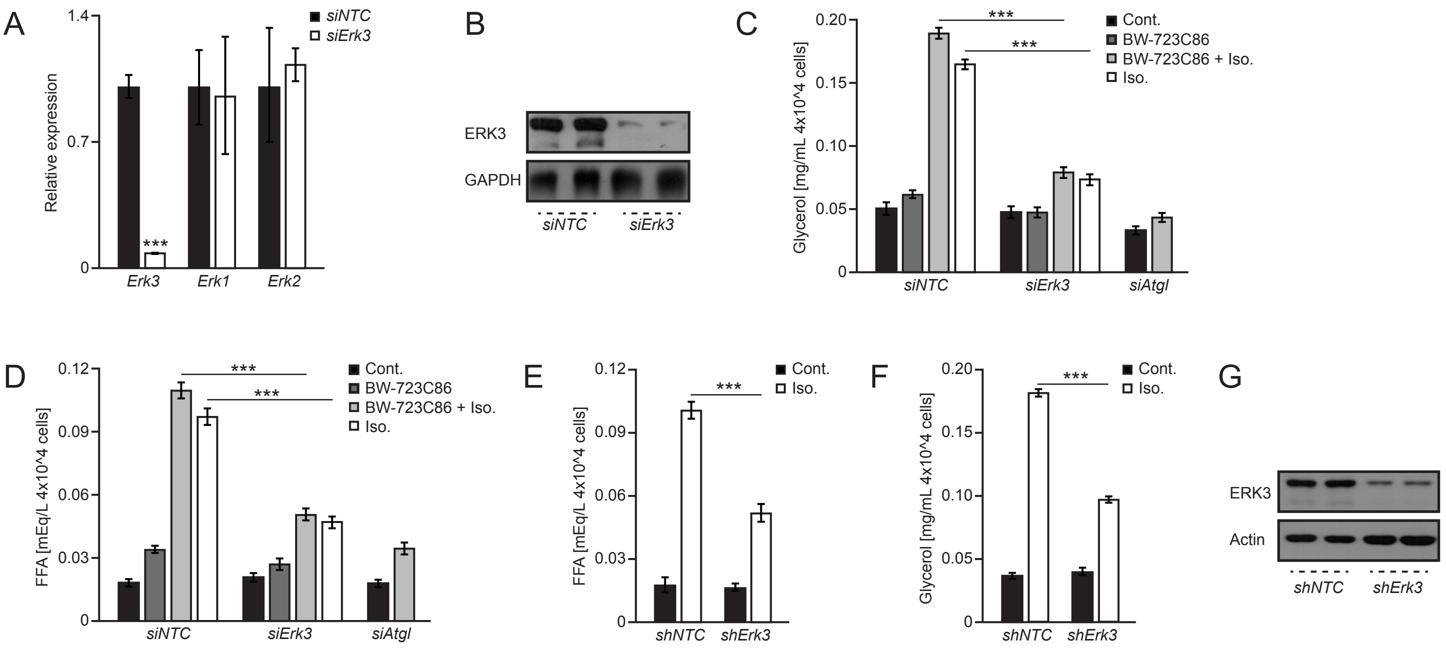

Supplemental Fig. S2 ERK3 promotes lipolysis in adipocytes. (a) Relative expression of indicated genes in adipocytes transfected with *Erk3*-specific or control NTC siRNA (n=3). (b) WB for ERK3 on extracts from 3T3L1 adipocytes transfected with specific siRNAs (n=2). (c) Glycerol and (d) FFAs output from 3T3L1 adipocytes transfected with specified siRNAs and stimulated as indicated for 2h (n=3). (e) FFAs and (f) glycerol release from *Erk3* shRNA transfected 3T3L1 adipocytes and control cells (n=3) stimulated as indicated for 2h. (g) WB analysis of ERK3 knockdown in 3T3L1 cells transfected with *Erk3* shRNA and control cells (n=2). For graphs - data presented average +/- SEM, *** P ≤0.001.

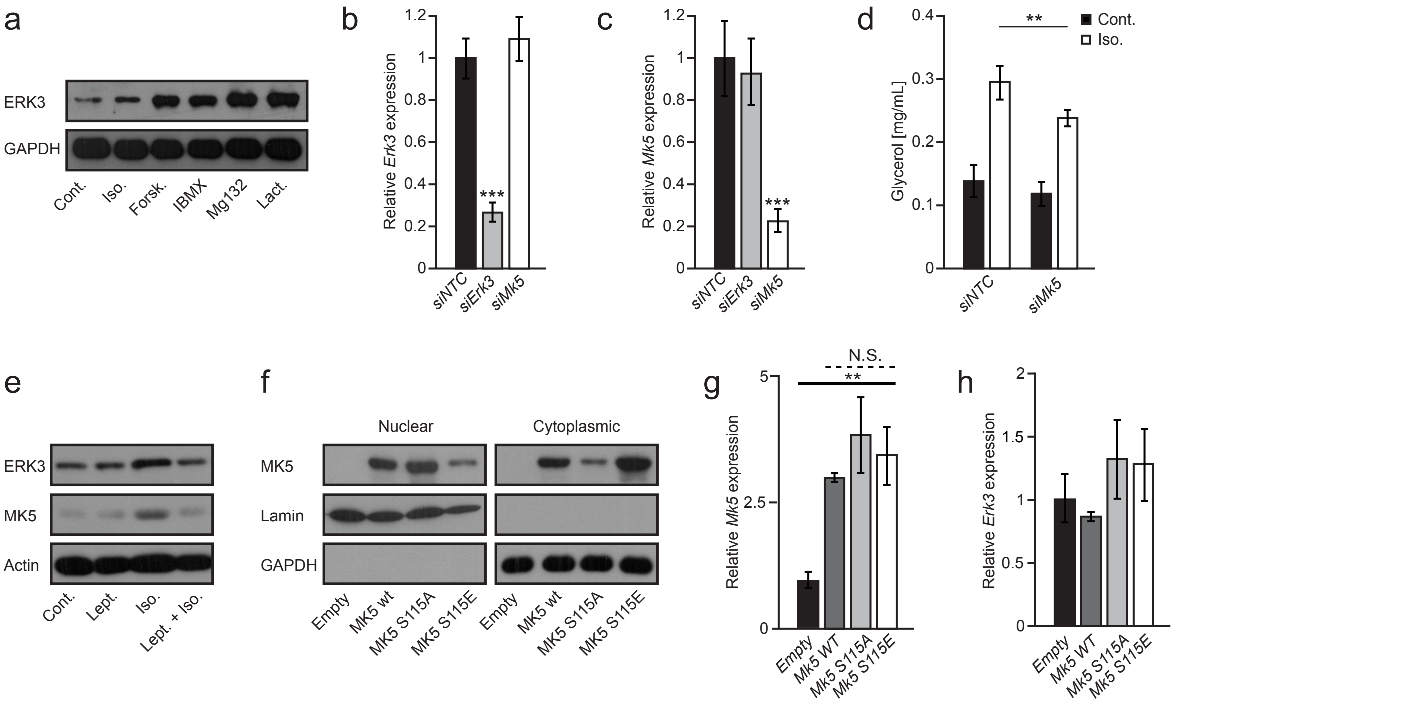

Supplemental Fig. S3 β-adrenergic signaling promotes ERK3 protein levels. (a) WB for ERK3 on undifferentiated 3T3L1 cells stimulated as indicated for 2h. (b) Relative expression of *Erk3* and (c) *Mk5* in 3T3L1 cells transfected with siRNA against *Erk3, Mk5* or *NTC*. (d) Glycerol release from control adipocytes and cells depleted from MK5 (si*Mk5*) stimulated with control medium or Iso. for 2h. (e) Indicated protein levels in 3T3L1 cells, stimulated as shown for 2h (Leptomycin B – Lept.). (f) The abundance of MK5 in the nucleus and cytoplasm of adipocytes expressing indicated MK5 mutants. Lamin and Gadph are positive controls for the nuclear and cytoplasmic fractions, respectively. (g) Relative expression of *Mk5* and (h) *Erk3* in 3T3L1 expressing indicated Mk5 mutants. n=3 for each experiment, for graphs - data presented average +/- SEM, ** P ≤0.01, *** P ≤0.001, N.S. - not significant

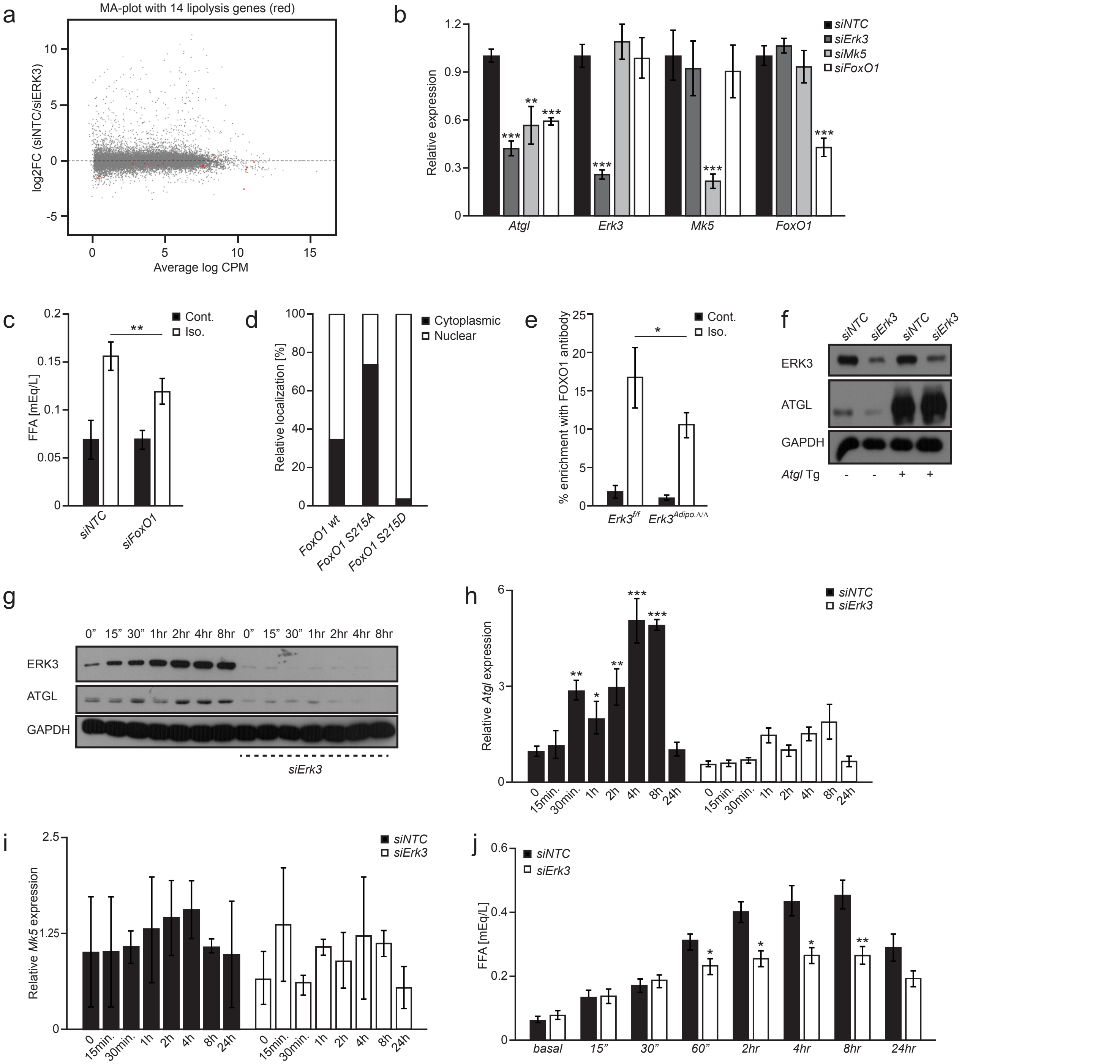

Supplemental Fig. S4 ERK3/MK5 promotes the expression of Atgl in the FOXO1-dependent manner. (a) MA-plot showing RNA-sequencing results. Differential expression and average abundance [indicated as log2 fold-change (FC) and log counts-per-million (CPM)] of all genes were calculated with EdgeR. Lipolysis-related genes are highlighted in red (n=4). (b) Relative expression of indicated genes in 3T3L1 cells depleted of specific proteins by siRNA. (c) FFA release from 3T3L1 cells transfected with FoxO1 or control siRNA and stimulated for 2h with isoproterenol (iso). (d) Relative localization of indicated FOXO1 mutants in the cytoplasmic and nuclear fraction of the adipocytes. (e) Relative enrichment of FOXO1 on the promoter of *Ucp1* in subWAT-derived adipocytes stimulated for 2h with iso. (f) WB for ERK3, ATGL, and GAPDH in cells overexpressing ATGL (Atgl tg) in which *Erk3* has been silenced. (g) WB for ERK3, ATGL, and GAPDH in 3T3L1 adipocytes with silenced *Erk3* and control cells stimulated for indicated time points with Iso. QPCR for *Atgl* (h) and *Mk5* (i) in 3T3L1 adipocytes with silenced *Erk3* and control cells stimulated for indicated time points with Iso. Release of FFAs (j) and glycerol (k) in 3T3L1 adipocytes with silenced *Erk3* and control cells stimulated for indicated time points with Iso. n=3 to 4 for each experiment, for graphs - data presented average +/- SEM, ** P ≤0.01, *** P ≤0.001

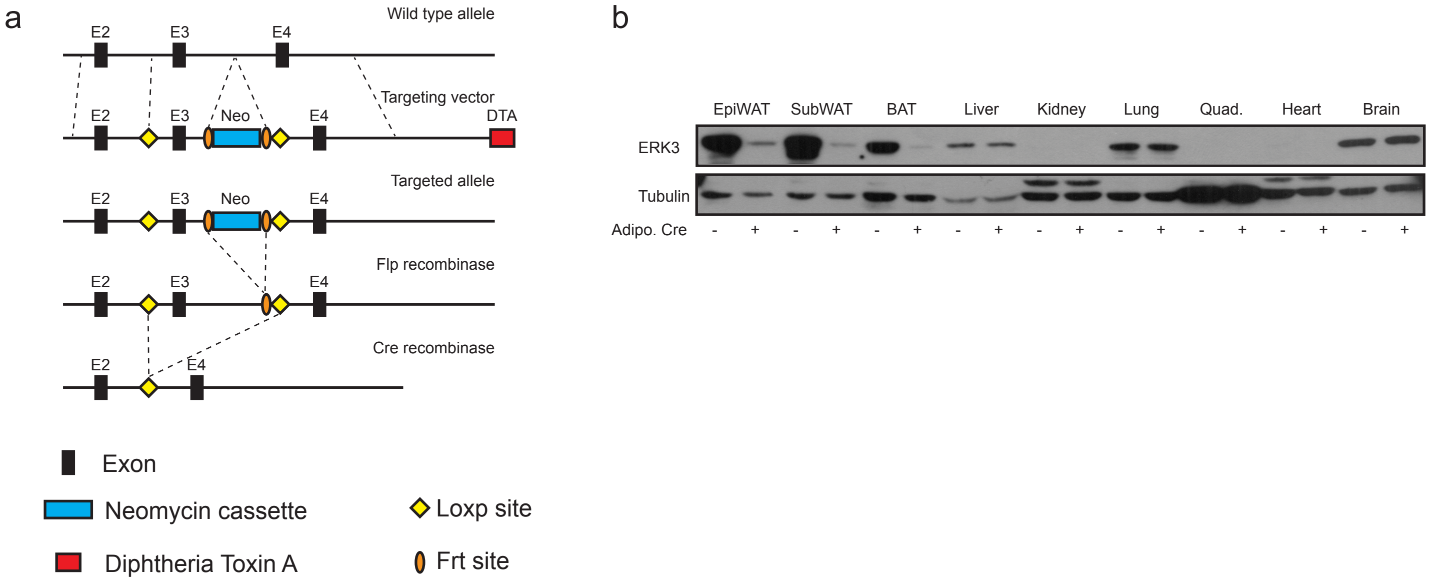

Supplemental Fig. S5 Generation of mice deficient for ERK3 specifically in adipocytes. (a) Schematic representation of the targeting strategy for the generation of Erk3-deficient mice. (b) WB confirming specific deletion of *Erk3* in different adipose tissue depots of *Erk3*$^{Adipo.\Delta/\Delta}$mice (n=3).

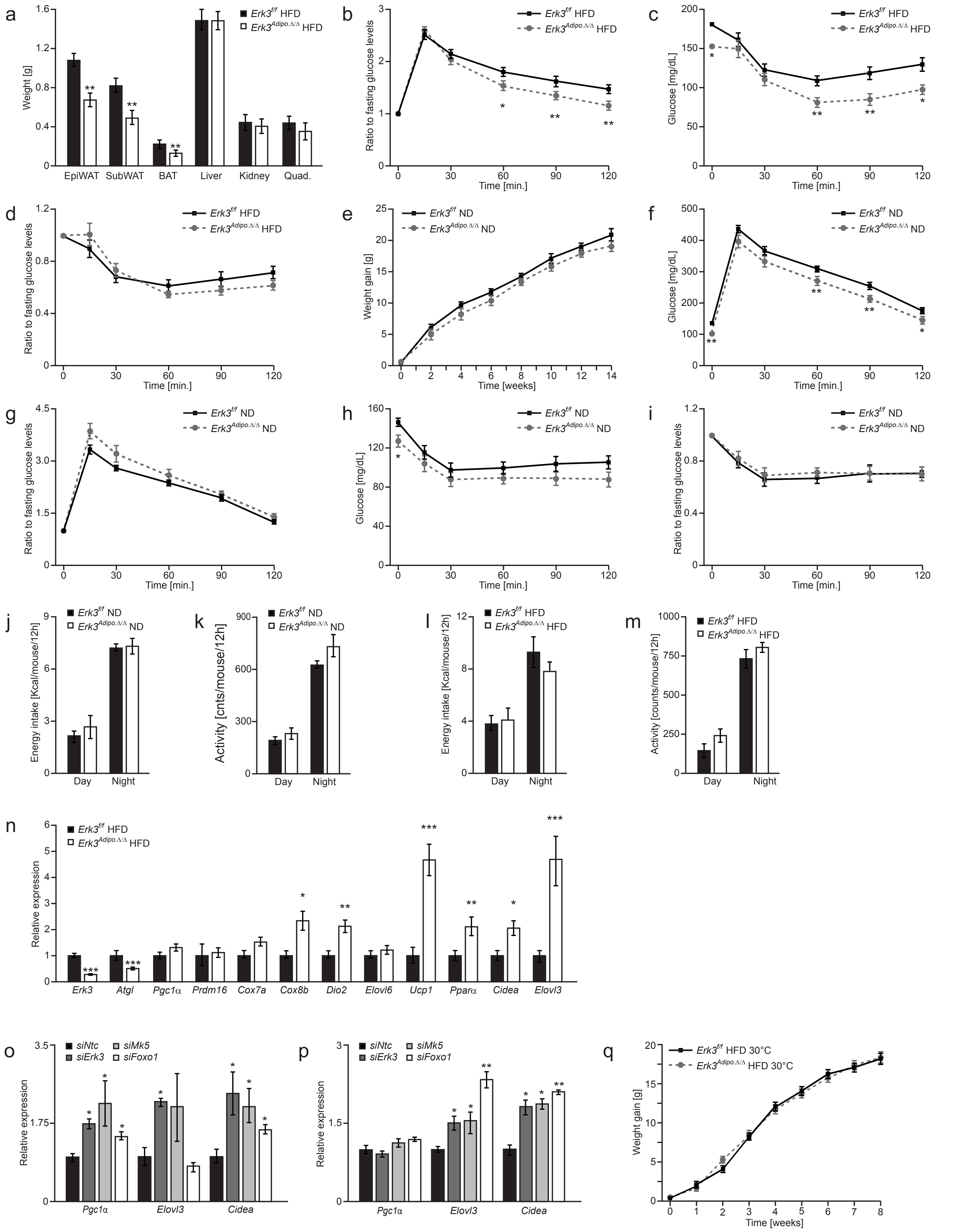
a
b
c
d
e
f
g
h
i
j
k
l
m
n
o
p
q
Erk3f/f HFD
Erk3Adipo.Δ/Δ HFD
Erk3f/f ND
Erk3Adipo.Δ/Δ ND
Erk3f/f HFD 30°C
Erk3Adipo.Δ/Δ HFD 30°C
siNtc
siErk3
siMk5
siFoxo1
Weight [g]
EpiWAT
SubWAT
BAT
Liver
Kidney
Quad.
Ratio to fasting glucose levels
Glucose [mg/dL]
Time [min.]
Weight gain [g]
Time [weeks]
Energy intake [Kcal/mouse/12h]
Activity [cnts/mouse/12h]
Activity [counts/mouse/12h]
Day
Night
Relative expression
Erk3
Atgl
Pgc1α
Prdm16
Cox7a
Cox8b
Dio2
Elovl6
Ucp1
Pparα
Cidea
Elovl3

Supplemental Fig. S6 Deletion of Erk3 in adipocytes protects against obesity-induced insulin resistance. (a) Indicated organ weights isolated from $Erk3^{Adipo.\Delta/\Delta}$ or respective control animals (n=11). (b) Glucose levels in relation to fasting glycemia during the glucose tolerance test (GTT) (n=11). Insulin tolerance test of $Erk3^{Adipo.\Delta/\Delta}$ and $Erk3^{f/f}$ mice fed a HFD for 13 weeks (n=11), represented in absolute values (c) and in relation to fasting glycemia (d). (e) Body weight evolution, glucose tolerance in absolute values (f) and in relation to fasting glycemia (g) and insulin tolerance in absolute values (h) and in relation to fasting glycemia (i) of $Erk3^{Adipo.\Delta/\Delta}$ and $Erk3^{f/f}$ mice fed normal diet (ND) for 14, 12 and 13 weeks respectively (n=8). (j) Energy intake and (k) voluntary activity of $Erk3^{Adipo.\Delta/\Delta}$ and $Erk3^{f/f}$ mice fed normal diet (ND). (l) Energy intake and (m) voluntary activity of $Erk3^{Adipo.\Delta/\Delta}$ and $Erk3^{f/f}$ mice fed HFD for 14 weeks (n=8). (n) Relative expression of indicated genes in brown adipose tissue from $Erk3^{Adipo.\Delta/\Delta}$ and $Erk3^{f/f}$ mice fed HFD (n=6). Relative expression of indicated genes in SVC-derived white (o) and brown (p) -derived adipocytes transfected with indicated siRNAs (n=4). (q) Body weight evolution of mice with indicated genotypes fed HFD and maintained at thermoneutral conditions. Data presented as average +/- SEM, * P ≤0.05, ** P ≤0.01, *** P ≤0.001

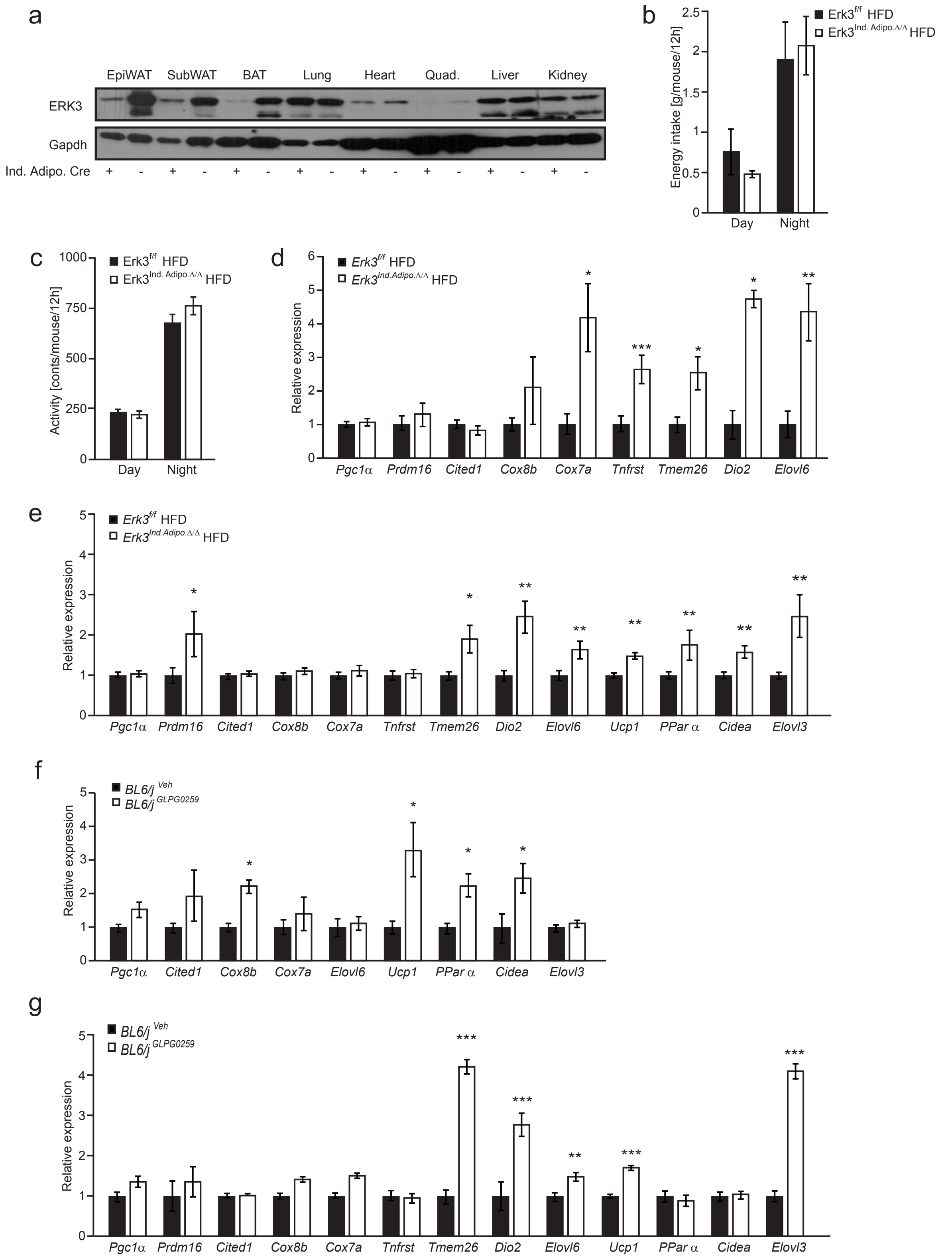

Supplemental Fig. S7 Deletion of ERK3 in obese mice does not affect food intake or motoric activity. (a) WB confirming specific deletion of ERK3 in different adipose tissue depots of *Erk3*$^{\text{Ind.Adipo.}\Delta/\Delta}$ mice (n=3). (b) Food and (c) voluntary activity of *Erk3*$^{\text{Ind.Adipo.}\Delta/\Delta}$ compared to respective *Erk3*$^{f/f}$ under HFD (n=8). Relative expression of indicated genes in (d) subWAT and (e) BAT isolated from *Erk3*$^{\text{Ind.Adipo.}\Delta/\Delta}$ mice fed HFD and corresponding control animals (n=8). Expression of the labeled genes in subWAT (f) and BAT (g) of mice treated with GLPG0259 inhibitor or vehicle for the period of one week (n=6). For graphs - data presented as average +/- SEM.
